# Rapid Adaptation and Interspecific Introgression in the North American Crop Pest *Helicoverpa zea*

**DOI:** 10.1093/molbev/msae129

**Published:** 2024-06-28

**Authors:** Henry L North, Zhen Fu, Richard Metz, Matt A Stull, Charles D Johnson, Xanthe Shirley, Kate Crumley, Dominic Reisig, David L Kerns, Todd Gilligan, Tom Walsh, Chris D Jiggins, Gregory A Sword

**Affiliations:** Department of Zoology, University of Cambridge, Cambridge CB2 3EJ, UK; Department of Entomology, Texas A&M University, College Station, TX 77843, USA; Bioinformatics and Biostatistics Core, Van Andel Institute, Grand Rapids, MI 49503, USA; AgriLife Genomics and Bioinformatics Service, Texas A&M University, College Station, TX 77843, USA; AgriLife Genomics and Bioinformatics Service, Texas A&M University, College Station, TX 77843, USA; AgriLife Genomics and Bioinformatics Service, Texas A&M University, College Station, TX 77843, USA; Animal and Plant Health Inspection Service, United States Department of Agriculture, College Station, TX, USA; Agrilife Extension, Texas A&M University, Wharton, TX, USA; Department of Entomology and Plant Pathology, North Carolina State University, Plymouth, NC, 27962, USA; Department of Entomology, Texas A&M University, College Station, TX 77843, USA; Animal and Plant Health Inspection Service, United States Department of Agriculture, Fort Collins, CO, USA; Black Mountain Laboratories, Commonwealth Scientific and Industrial Research Organization, Canberra, Australia; Department of Zoology, University of Cambridge, Cambridge CB2 3EJ, UK; Department of Entomology, Texas A&M University, College Station, TX 77843, USA

**Keywords:** introgression, *Helicoverpa zea*, *Helicoverpa armigera*, selective sweep, isolation by distance, pesticide resistance, Bt resistance, population genomics, pests, invasive species

## Abstract

Insect crop pests threaten global food security. This threat is amplified through the spread of nonnative species and through adaptation of native pests to control measures. Adaptations such as pesticide resistance can result from selection on variation within a population, or through gene flow from another population. We investigate these processes in an economically important noctuid crop pest, *Helicoverpa zea*, which has evolved resistance to a wide range of pesticides. Its sister species *Helicoverpa armigera*, first detected as an invasive species in Brazil in 2013, introduced the pyrethroid-resistance gene *CYP337B3* to South American *H. zea* via adaptive introgression. To understand whether this could contribute to pesticide resistance in North America, we sequenced 237 *H. zea* genomes across 10 sample sites. We report *H. armigera* introgression into the North American *H. zea* population. Two individuals sampled in Texas in 2019 carry *H. armigera* haplotypes in a 4 Mbp region containing *CYP337B3*. Next, we identify signatures of selection in the panmictic population of nonadmixed *H. zea*, identifying a selective sweep at a second cytochrome P450 gene: *CYP333B3*. We estimate that its derived allele conferred a ∼5% fitness advantage and show that this estimate explains independently observed rare nonsynonymous *CYP333B3* mutations approaching fixation over a ∼20-year period. We also detect putative signatures of selection at a kinesin gene associated with Bt resistance. Overall, we document two mechanisms of rapid adaptation: the introduction of fitness-enhancing alleles through interspecific introgression, and selection on intraspecific variation.

## Introduction

Insect pests destroy 5% to 20% of major grain crop production, and losses are set to increase substantially over coming decades as a result of climate change, the evolution of pesticide resistance, and the spread of invasive species via international trade routes (Paini et al. 2016; Deutsch et al. 2018; Gould et al. 2018). Understanding the role that evolution plays in the ecology of pests and their outbreaks is therefore a priority (Green et al. 2020; Luke et al. 2023). The short generation times and large effective population sizes of many invertebrate pest species results in high rates of molecular evolution and rapid allele frequency shifts in response to natural selection (Petit and Barbadilla 2009; Thomas et al. 2010). At the same time, extreme selective regimes, such as those imposed by pesticide exposure, can result in rapid adaptation (Hawkins et al. 2019). The strong dispersal ability of many insect pests, especially in the context of contiguous habitat in monoculture, means that pest populations often exist as highly connected metapopulations that cover agricultural landscapes (Mazzi and Dorn 2012). As a consequence, fitness-enhancing alleles can readily spread across space (McDonald and Linde 2003). Human activity can also mediate dispersal at larger geographic scales (i.e. across agricultural systems and continents) such that insecticide resistance can rapidly arise through gene flow—a process significantly faster than adaptation from de-novo mutation (Tay and Gordon 2019). The extent of global trade networks means that many closely related pest species come into secondary contact, opening up the possibility of adaptive introgression not only between populations, but also between divergent ecotypes and species (Song et al. 2011; Valencia-Montoya et al. 2020). Together these factors enhance the evolutionary potential of insect pests, with two key consequences. First, adaptive responses can occur at timescales relevant to year-to-year pest management strategies. Second, the global connectedness of many pest populations means that such strategies must be multilateral. Large-scale genomic monitoring is widely accepted as a promising emerging means of informing management action to address these consequences.

Population genetics has long been used as a tool for quantifying evolutionary change in agricultural pests, especially with respect to insecticide resistance (Mallet 1989). The additional information and contiguity of resolution offered by genome-resequencing data have created renewed interest in this field, and genomic approaches are clearly emerging as a key tool for monitoring pest populations under both proactive and reactive management plans (Hamelin and Roe 2020; Neafsey et al. 2021; North et al. 2021; Sherpa and Després 2021). Recent studies have demonstrated the use of population genomics approaches to define management units by quantifying population connectivity (Chen et al. 2021; Paris et al. 2022), identifying loci associated with the evolution of pesticide resistance by inferring the action of selection (Love et al. 2023), and reconstructing the spatial spread of species or alleles of interest (Tay et al. 2022). With appropriate analysis and sampling design, population genomics can be used to extract otherwise-inaccessible biological information to understand the evolutionary history of pest populations and inform management plans.

The corn earworm *Helicoverpa zea* is a polyphagous noctuid moth common throughout the Americas. A notorious pest of maize and cotton, *H. zea* is one of the most economically significant crop pests in the agricultural powerhouses of Brazil and the United States (Fitt 1989; Cunningham and Zalucki 2014; Olmstead et al. 2016a, 2016b; Cook and Threet 2019; Olivi et al. 2019). Although maize is its dominant host plant, larvae are known to feed on at least 122 other species, of which 29 are major crops including wheat, soy, rice, sorghum, and tomato (Cunningham and Zalucki 2014). Larvae tend to feed on the fruiting body of the plant, thereby directly damaging produce (Luttrell & Jackson 2012). Generation times vary depending on latitude (5 to 10 generations/year at lower altitudes), though facultative diapause enables pupa to persist underground up to at least 40°N during winter (Hardwick 1965; Parajulee et al. 2004; Morey 2012). Adults are highly effective long-distance dispersers, expanding northward into extensive areas of maize production during summer to a latitude of ∼52°N in flights large enough to detect using ground-based radar (Westbrook 2008; Jones et al. 2018). The species’ range is expected to expand 2-fold by 2099 as warmer winters reduce the number of lethal low-temperature events (Lawton et al. 2022). Repeated admixture due to seasonal re-establishment from southern populations, combined with long-range dispersal over highly connected agricultural habitat, result in a highly connected, and genetically diverse metapopulation in the north (Margosian et al. 2009; Seymour et al. 2016).

Multiple studies have found that the local distribution of crops producing *Bacillus thuringiensis* (Bt) toxins in a given year can predict *H. zea* damage in subsequent years (Arends et al. 2021, 2022). This observation implies that selection results in geographically localized phenotypic change between generations, so parent–offspring dispersal should primarily occur at the same geographic scale. In contrast, population genetics studies have reported effective panmixia across the North American range, though there is mixed evidence for this observation, and the only study to employ whole-genome data compared just two sample sites (Seymour et al. 2016; Perera et al. 2020; Taylor et al. 2021). Increased sampling effort—in terms of both geographic range and number of loci—can reveal population structure that is otherwise undetectable, as demonstrated in studies of *Helicoverpa armigera* (Zhang et al. 2022; Jin et al. 2023). Characterizing the landscape of effective migration is key to understanding how rapidly adaptive variants underlying pesticide resistance can spread.

*Helicoverpa zea* has evolved resistance to several pesticides. In the United States, the organochlorine DDT was effective for *H. zea* control from its post-war implementation until the 1960s; resistance to methyl parathion (an organophosphate) introduced 1960s was initially detected in the 1970s; several pyrethroids introduced in the 1970s were effective until resistance started to become apparent in the 1990s and 2000s (Abd-Elghafar et al. 1993; Walsh et al. 2022). The specific mode of action of these pesticides means that resistance phenotypes could in many cases be underlain by mutations at one or few loci and therefore evolve rapidly in response to strong selection (Ibrahim et al. 2015). As a member of the “Megapest” genus of polyphagous herbivores, *Helicoverpa*, *H. zea* may be particularly well equipped to evolve resistance due to its ecology and its capacity to metabolize a wide array of host plant defences (Mallet 1989; Gordon et al. 2010; Bras et al. 2022). Although many phenotypes with different genetic architectures can underly resistance to a given pesticide, certain gene families have been repeatedly implicated as targets of selection. This is true of cytochrome P450 genes involved in xenobiotic metabolism not only in *Helicoverpa* but in pest species spanning the tree of life (Kreiner et al. 2019; Nauen et al. 2022).

By the 1990s, it became clear that the evolution of resistance was outpacing the development of novel pesticides, highlighting the need for a strategic shift toward integrated pest management. New approaches implemented for lepidopteran pests included the use of cotton and maize crops engineered to produce Bt toxins, which today constitute 82% of US maize crops and 88% of cotton (Reisig et al. 2022). In contrast to the nerve- and muscle-targeting insecticides discussed above, Bt toxins induce pore formation in the midgut membrane. Resistance to these toxins therefore requires selection on a different set of loci. The most successful management plans made use of Bt crops expressing multiple toxins (i.e. different Cry or Vip proteins) at high concentrations (>25 × the dose required to kill susceptible insects) planted among non-Bt refuges in which rare resistant individuals can reproduce with susceptible mates. For insect pests generally, this approach was broadly successful at minimizing unidirectional selection pressures, reducing net pesticide use and slowing the rate of resistance evolution. However, *H. zea* is among a few pest species to have evolved Bt resistance in the field. Cry1Ac resistance was reported in the early 2000s, at which point multitoxin crops additionally expressing Cry2 were planted (Ali et al. 2006). By 2016, resistance to Cry1 toxins had become common throughout the US and Cry2 resistance was emerging (Dively et al. 2016; Reisig et al. 2018). Recent studies concluded that resistance to Cry2Ab2 has become common, and that selection was ongoing as of 2019 (Yu et al. 2021; Huang et al. 2023). The genetic basis of Bt resistance is known to be complex and likely arose from standing variation, with unique genetic architectures underlying resistance to different Cry toxins (Taylor et al. 2021; Benowitz et al. 2022).

In addition to intraspecific adaptation, a major concern for the spread of pesticide resistance in North American *H. zea* is through interspecific introgression from its sister species *H. armigera*. Commonly known as the cotton bollworm, *H. armigera* has a broad Afro-Eurasian native range. Among the most economically damaging crop pests in the world, *H. armigera* is more polyphagous and resistant to a substantially broader array of pesticides compared with *H. zea* (Cunningham and Zalucki 2014). *H*. *armigera* was first detected in Brazil in 2013, where the two species hybridized (Tay et al. 2013; Anderson et al. 2018; Ivey and Hillier 2023). This resulted in the adaptive introgression of the *CYP337B3* gene into South American *H. zea* populations (Valencia-Montoya et al. 2020). *CYP337B3*, otherwise absent in *H. zea*, is the result of unequal crossover from two other cytochrome P450 genes and confers fenvalerate resistance (Joußen et al. 2012). The variant has arisen multiple times in *H. armigera*, and can encode resistance to various pyrethroids including cypermethrin and deltamethrin (Rasool et al. 2014; Durigan et al. 2017).

*Helicoverpa armigera* is now established throughout much of South and Central America. The risk of *H. armigera*, or admixed *H. armigera-zea* individuals, spreading into suitable North American habitat is considered high, and such an event would put at risk US crop production valued at $USD 78 billion per annum (Kriticos et al. 2015). *H. armigera* has been intercepted at US ports more than 1,000 times, suggesting that introduction via shipping routes from its broad Afro-Eurasian range is also a substantial risk (Kriticos et al. 2015). Since the two species are difficult to distinguish phenotypically, and because of the extent of interspecific admixture in South America, the detection of invasive *H. armigera* alleles into North America requires genetic surveillance of native *H. zea* populations. To date, there have been no published reports in the scientific literature of *H. armigera* establishing on the North American mainland. In 2015, three specimens captured in Florida carried the *H. armigera* COI haplotype (Tembrock et al. 2019), and multiple adults were captured in areas adjacent to Chicago O’Hare International Airport (USDA APHIS 2024), though these are isolated incidents. There have been unpublished reports of *CYP337B3* detected in *H. zea* survey samples, though this observation may result from parallel evolution, as observed in *H. armigera* (Rasool et al. 2014).

**Fig. 1.**
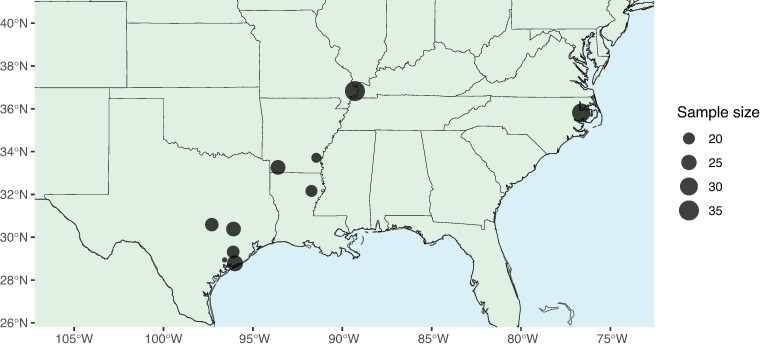
Individual sampling effort at 10 sites in 2019. Sample site information is detailed in supplementary table S1, Supplementary Material online.

Here, we use a population genomics approach to (i) test for signatures of introgression from *H. armigera* into North American *H. zea*, (ii) characterize effective migration across space to understand how rapidly resistance alleles may spread, and (iii) conduct a genome-wide scan for evidence of selective sweeps at known pesticide and Bt-resistance loci. To achieve this, we resequenced the genomes of 237 *H. zea* individuals across 10 locations in the United States collected in 2019 (see Materials and Methods) (Fig. 1).

## Results

### Evidence for Introgression of Pesticide Resistance Genes into North American *H. zea*

**Fig. 2.**
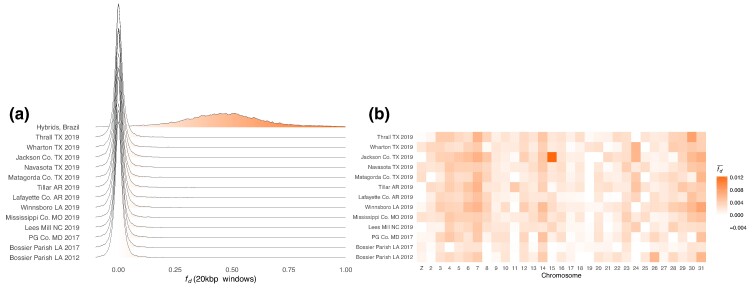
No evidence of *H. armigera* in *H. zea* except for on chromosome 15 among a subset of *H. zea* individuals collected in Jackson County, TX, in 2019. a) Distribution of $\hat{\;f_{d}}$ calculated in 20 kbp windows where P1: *H. zea* sampled in 2002, P3: *H. armigera*, outgroup: *H. punctigera*. The statistic was calculated for 14 different P2 sets: 10 sets of *H. zea* samples collected at different sites in 2019, 2 sets of *H. zea* collected in 2017, one set of *H. zea* collected in 2012, and a positive control of 9 individuals sampled in Brazil shown to be admixed individuals carrying both *H. armigera* and *H. zea* ancestry. b) Data presented as the mean per chromosome. *H. zea* samples collected in 2002, 2012, and 2017 are from Taylor et al. (2021).

We tested for the presence of *H. armigera* ancestry by calculating $\hat{\;f_{d}}\;$ in 20 kbp windows for individuals from each sample, using *H. zea* samples collected in Louisiana in 2002 as representative nonadmixed samples and *H. punctigera* as an outgroup. We repeated this test for hybrids sampled in Brazil used as a positive control. The distribution of $\hat{\;f_{d}}\;$ was centred on zero for all test sets apart from the positive control (Fig. 2a). The same result is seen in samples reported in Taylor et al. (2021), collected in the United States in 2012 and 2017. These results indicate balanced proportions of ABBA and BABA patterns, indicating little to no introgression at any sample site, at least compared to the magnitude of introgression seen in Brazil. However, $\hat{\;f_{d}}\;$ is elevated on chromosome 15 among samples collected in 2019 in Jackson County, TX (Fig. 2b). Computing $\hat{\;f_{d}}\;$ for each of the 16 samples from this site individually shows that the pattern is driven by extreme values of $\hat{\;f_{d}}\;$ in only two individuals (supplementary fig. S1, Supplementary Material online).

**Fig. 3.**
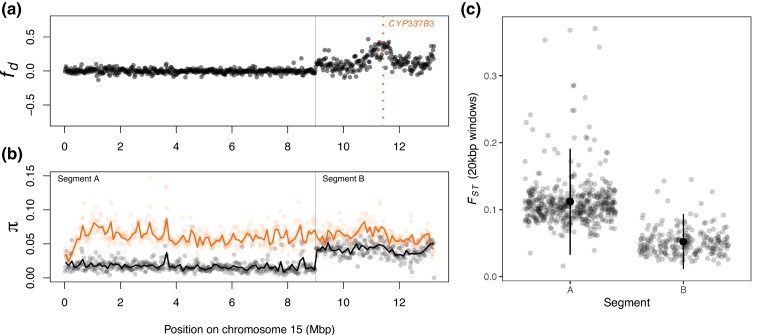
Introgression of *CPY337B3.* a) $\hat{\;f_{d}}$ calculated in 20 kbp windows along chromosome 15, where P1 is *H. zea* sampled in 2002, P2 are Ja15 and Ja25 in supplementary fig. S1, Supplementary Material online, P3 is *H. armigera*, and the outgroup is *H. punctigera*. b) Nucleotide diversity (*π*) calculated in 20 kbp windows (points) and 100 kbp windows (lines) for the admixed individuals (Ja15 and Ja23; black) and for *H. armigera* (coloured). c) Genetic differentiation ($F_{ST}$), calculated in 20 kbp windows, between the admixed individuals and *H. armigera* in the chromosomal segments labeled in (b). *H. zea* samples collected in 2002 are from Taylor et al. (2021) and samples from other *Helicoverpa* species are from Anderson et al. (2018); see supplementary table S2, Supplementary Material online for details.

For those individuals, $\hat{\;f_{d}}\;$ peaks at the *CYP337B3* locus (Fig. 3). Nucleotide variation for these individuals is reduced across most of Chromosome 15 relative to *H. armigera*, but matches *H. armigera* in a terminal ∼4 Mbp region around *CYP337B3*. Given that the effective population size of *H. armigera* is twice that of *H. zea* (Anderson et al. 2018), this pattern suggests *H. armigera* ancestry dominates in this region. Based on patterns $\hat{\;f_{d}}\;$ and *π*, we defined two segments of chromosome 15: A (0 to 9 Mbp), which shows no signs of admixture, and B (>9 Mbp) in which *H. armigera* haplotypes have introgressed.

We reasoned that if segment B consists of largely *H. armigera* ancestry, genetic differentiation, and divergence from *H. armigera* should be lower in this region. Segment B shows reduced genetic differentiation relative to *H. armigera* ($\bar{F_{ST}}$ = 0.05 compared to 0.11 in Segment A; *P* < 0.01, *t* = 25.606, df = 656, Welsch two-sample *T* test; Fig. 3c). Genetic divergence also differed by segment ($\bar{d_{xy}}$ = 0.086 and 0.073 respectively; *P* < 0.01, *t* = 10.495, df = 515.7; supplementary fig. S2, Supplementary Material online); this difference was less pronounced, as expected given that genetic divergence between species should accumulate slower than differentiation.

**Fig. 4.**
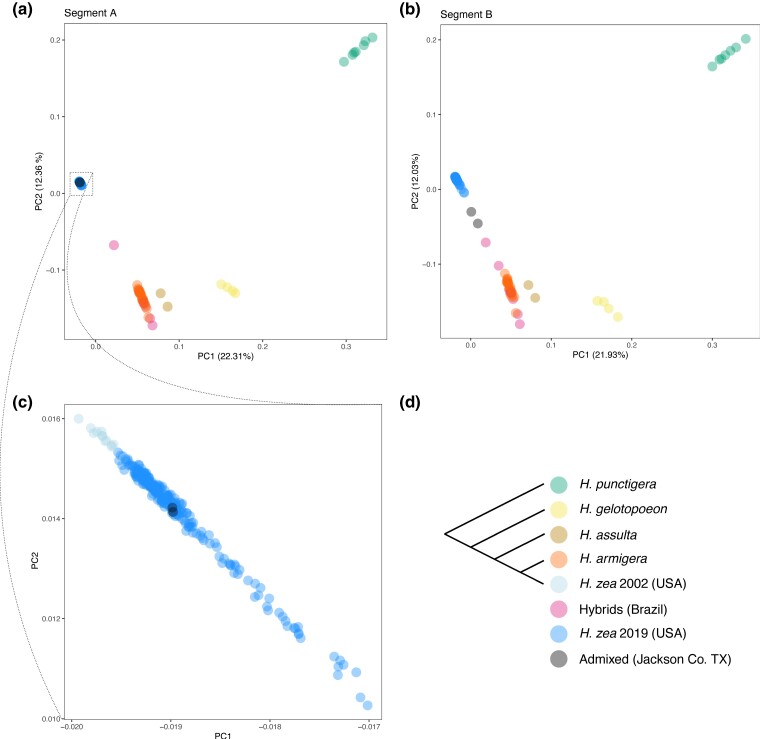
Visualizing local ancestry with principal components analysis. a,b) Principal components 1 and 2 calculated using SNPs form segments A and B of chromosome 15 (see Fig. 3). In Segment B, the two admixed samples (black points) are closer to *H. armigera* samples and admixed samples from Brazil. c) Within segment A, the two admixed sales cluster with other *H. zea* samples collected in 2019. d) Consensus species tree. *H. zea* samples collected in 2002 are from Taylor et al. (2021) and samples from other *Helicoverpa* species are form Anderson et al. (2018); see supplementary table S2, Supplementary Material online for details.

These differences were apparent when visualizing the data with principal components analysis (PCA). At segment A, the two admixed individuals cluster completely within other *H. zea* samples from 2019 in principal component space, whereas at segment B the individuals are closer to known Brazilian hybrid samples (Fig. 4).

We next sought to determine whether the admixed samples carried the *H. armigera CYP337B3* gene, and if so, to use the gene tree to identify a potential *H. armigera* source population. This is because *CYP337B3* has arisen independently in multiple *H. armigera* populations (Rasool et al. 2014). To investigate this, we reconstructed a maximum likelihood gene tree at the *CYP337B3* locus (HaChr15:11436565-11440168, as mapped by Anderson et al. 2018 and used by Valencia-Montoya et al. 2020), comparing the admixed samples to publicly available *H. armigera* samples representing the breadth of the species’ phylogeographic diversity. The two admixed individuals form a clade with *H. armigera* samples at the *CYP337B3* locus (supplementary fig. S4 to S5, Supplementary Material online). In 100% of bootstrap iterations, nonadmixed *H. zea* samples were split from the clade consisting of admixed individuals and *H. armigera* samples. An improved phylogeny generated using a constrained topology revealed two clear lineages within the *H. armigera* clade (Cades 1 and 2 in supplementary fig. S6, Supplementary Material online).

Together, these results suggest that two of 237 individuals sampled are admixed, that introgression in these two samples is concentrated around the *CYP337B3* locus, and that both samples carry at least one *H. armigera CYP337B3* allele. Given the gene's semidominant mode of action, it is likely that both individuals showed some degree of resistance to fenvalerate (Joußen et al. 2012).

### High Connectivity Across the North American *H. zea* Metapopulation

**Fig. 5.**
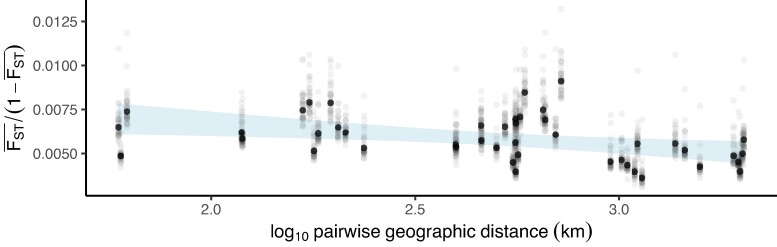
Low genetic differentiation irrespective of geographic distance among nonadmixed *H. zea*. Pairwise genetic differentiation regressed on pairwise geographic distance for each of 45 possible comparisons among 10 sample sites. Gray points are results for each of 31 chromosomes; black points are autosome-wide averages. Genetic differentiation was calculated as mean $F_{ST}$ across 20 kbp windows. Confidence intervals show standard error around the linear model. There is no signal of isolation-by-distance.

We next sought to characterize the extent of allele sharing among nonadmixed samples across the landscape to better understand how rapidly adaptive alleles (including those introduced from *H. armigera*) could spread. There was no positive relationship between geographic distance and genetic differentiation between sampling sites (one-sided Pearson's product moment correlation for genome-wide average $F_{ST}$: *t* = −2.8132, 43 df, *P* = >0.05), and the overall level of genetic differentiation was very low between sample sites ($F_{ST}$ < 0.01), so the North American *H. zea* metapopulation shows no signs of isolation by distance (Fig. 5). A two-tailed correlation test revealed that the relationship between genetic differentiation and geographic distance was slightly negative (*P* = 0.005277, *t* = −2.939, intercept = 6.528e−03, slope = −1.017^−6^), though the magnitude of the gradient was so negligible as to be biologically meaningless (−0.01017 $\Delta F_{ST}\;$ per 10^4^km). Samples did not cluster by geography when plotting autosomal loci using PCA, and *faststructure* showed that K=1 maximized the marginal-likelihood and best-explained population structure (supplementary fig. S8 to S9, Supplementary Material online). Since we saw no signs of population structure genome-wide, sites segregating among samples from all locations could be used to detect selective sweeps. This provided us with substantial statistical power for our third aim.

### Signatures of Selection Throughout the Genome

Given the absence of population structure among our samples, we included all individuals in a genome-wide scan for selective sweeps. We identified putative sweeps as extreme outliers for the composite likelihood ratio (CLR) implemented in *SweepFinder2* (see Materials and Methods). Three sweeps occurred on chromosomes 13, 15, and 25 (Figs. 7 and 8; supplementary fig. S10, Supplementary Material online). The sweep on chromosome 15 occurred within Segment A (Fig. 3) and showed no signs of introgression from *H. armigera* in any samples.

**Fig. 6.**
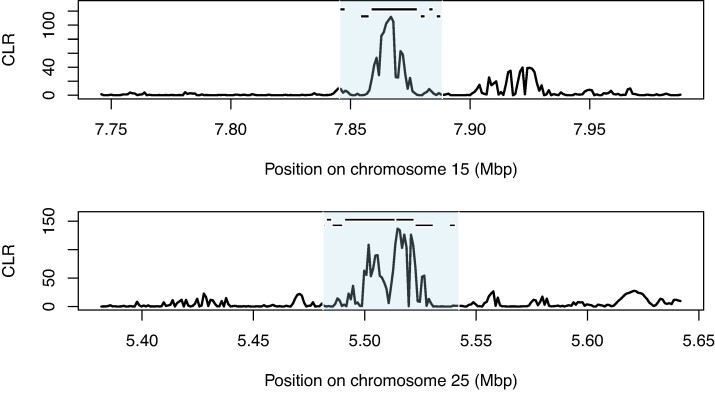
Sweep regions on chromosomes 15 and 25. CLR calculated in *SweepFinder2*. Black bars indicate the position of gene annotations on the forward strand (top) and reverse strand (bottom) listed in supplementary table S3, Supplementary Material online. Shaded area indicates the sweep region.

**Fig. 7.**
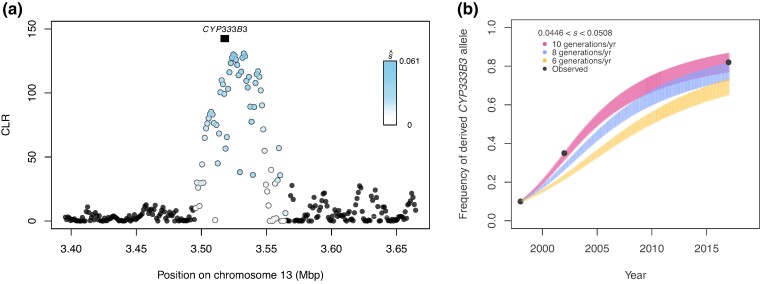
Selective sweep at *CYP333B3.* a) CLR calculated in *SweepFinder2*, with points colored by their estimated selection coefficient. Black box indicates the position of *CYP333B3*. b) Predicted frequency of a dominant-acting derived *CYP333B3* allele for each generation given the estimated selection coefficient at that locus assuming 6, 8, and 10 generations per year. For each generation, a vertical line extends from the retrodicted allele frequency under the lower estimate of the selection coefficient ($\hat{s}$ = 0.0446) to that estimated under the upper estimate ($\hat{s}\;$ = 0.0508). Line colors correspond to assumed generation time. Black points are independently estimated allele frequencies from Taylor et al. (2021).

Several genes within sweep regions have potential roles in immune response and pesticide resistance (supplementary table S4, Supplementary Material online). The sweep on chromosome 15 (Fig. 6) contained six unique gene annotations, with the vast majority of CLR outliers observed within one gene—*Hyd*-like—encoding an immune-associated E3 ubiquitin-protein ligase (Cammarata-Mouchtouris et al. 2020). The sweep on chromosome 25 contained 7 functional annotations (Fig. 6) and the sweep on chromosome 13, contained two genes including a different cytochrome P450 gene, *CYP333B3* (supplementary table S3, Supplementary Material online; Fig. 7).

### Evidence of Strong, Recent Selection at *CYP333B3*

The largest sweep occurred at the cytochrome P450 gene *CYP333B3* on chromosome 13. This gene is of interest because of its association with pesticide resistance in *Helicoverpa* (de la Paz Celorio-Mancera et al. 2011; Yangchun 2014; Amezian et al. 2021; Shi et al. 2022), and because nonsynonymous substitutions in this gene are known to have increased in frequency in North American *H. zea* (Taylor et al. 2021). Assuming direct selection at this locus, we estimate the selection coefficient is in the range 0.0446 < $\hat{s}\;$ < 0.0508 (Fig. 7a). This estimate is based on estimates of the effective population size, recombination rate, and qualities of the sweep (see Materials and Methods for discussion of our simplifying assumptions and sources of error).

In North American *H. zea*, Taylor et al. (2021) identified temporal genetic differentiation concentrated in a region that tightly overlapped with the sweep we observed on chromosome 13. In the same study, the authors were able to go back to *H. zea* samples from 1998, 2002, and 2017 in their freezer collection to genotype individuals at this locus, showing that the proportion of individuals with derived nonsynonymous *CYP333B3* mutations had increased over time. This afforded us an opportunity to determine whether our estimate of the selection coefficient was consistent with the change in allele frequency that they observed.

Allele frequencies generated using our estimate of $\hat{s}$ closely matched frequencies measured by Taylor et al. when we assumed a dominant mode of inheritance and a biologically realistic generation time of 8 to 10 generations per year (Fig. 7a). We also fit an estimate of the selection coefficient (${\hat{s}}_{\;fit}$) and dominance coefficient (${\hat{h}}_{\;fit}$) to the allele frequencies empirically estimated by Taylor et al. The dominance coefficient that best explained the data were >0.997 regardless of generation time when jointly optimized with the selection coefficient, consistent with our assumption of complete dominance. When dominance was assumed, $\;{\hat{s}}_{\;fit}$ closely matched the $\hat{s}\;$ range for 10 generations/yr, but was generally higher for slower generation times (supplementary table S7, Supplementary Material online).

However, the two selection coefficient estimates differed by less than 0.05 for generation times between 5 and 10 generations/year (supplementary table S7, Supplementary Material online). Therefore, we were able to approximate the strength of selection at this locus from a single-timepoint sample. Moreover, this approximation was accurate enough to infer that anthropogenic selection led to the near-fixation of a derived *CYP333B3* allele within ∼20 year (*q* = 0.956 in 2019; supplementary table S6, Supplementary Material online).

Patterns of genetic differentiation and diversity confirm that the selected allele arose within *H. zea* and was subject to selection within the last 20 years. When comparing *H. zea* samples from 2002 and 2019, genetic differentiation is greatest at the *CYP333B3* locus but otherwise low (supplementary fig. S11A, Supplementary Material online), and at the same locus there is a strong excess of homozygous haplotypes among 2019 samples (supplementary fig. S12, Supplementary Material online). Both of these results are consistent with a selective sweep occurring primarily in the intervening time. By contrast, differentiation is high across the full extent of chromosome 13 when comparing 2019 *H. zea* samples to *H. armigera*—especially at the *CYP333B3* locus (supplementary fig. S11B, Supplementary Material online). Among the 2019 samples, the average rate of coalescence was elevated at the locus. Specifically, there was a dearth of genealogies coalescing >2 $N_{e}$ generations in the past at *CYP333B3* (supplementary fig. S11C, Supplementary Material online) again consistent with the effects of a selective sweep. These results contradict expectations under adaptive introgression, which would leave deep coalescence events at loci abutting the selected gene (Setter et al. 2020). On chromosome 13, the average height of coalescent genealogies estimated within individuals was 2 $N_{e}$ generations (SE 1.5 × 10^−4^  $N_{e}$ generations), contrary to what we would expect if some sampled haplotypes originated in a divergent population or species (see Materials and Methods). Together, these results indicate a selective sweep resulting from strong selection for a derived *CYP333B3* allele over the past 20 years, and rule out introgression of *CYP333B3* from *H. armigera*.

Relative differences in haplotype homozygosity were minimal when comparing samples from different geographic regions, as opposed to different time-points (supplementary fig. S13 to S17, Supplementary Material online). However, marginal differences in haplotype homozygosity between samples from different geographic regions are apparent at the *CYP333B3* locus (supplementary fig. S14 to S17, Supplementary Material online). Variation in *CYP333B3* allele frequency matches this pattern (compare supplementary table S6, Supplementary Material online to supplementary fig. S14 to S17, Supplementary Material online). These results suggest that the sweep was close to fixation in 2019 but not yet complete—the exception being North Carolina, where the allele is fixed and the signal of selection is clearest.

### Signatures of Selection at Candidate Bt-Resistance Genes

**Fig. 8.**
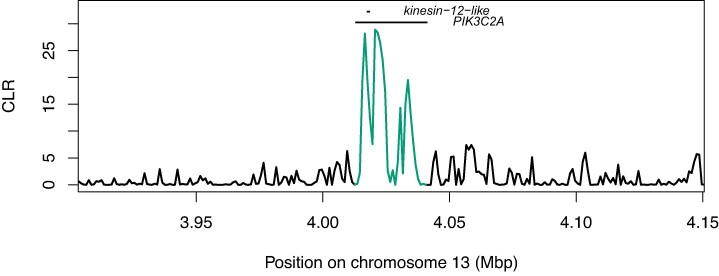
Selective sweep likelihood ratio at the locus *PIK3C2A/kinesin-12*-like. CLR values in the region of the loci *PIK3C2A* and *kinesin-12*-like, encoded on opposing strands. Both genes are candidate Cry1Ac resistance loci. Values overlapping with the annotation positions are highlighted.

None of the candidate Bt resistance loci that we mapped occurred in sweep regions, however one candidate locus on chromosome 13 occurred within the upper 1st percentile of all CLR values (Fig. 8). This locus (chr13:4012595-4041462) includes *PIK3C2A* and *kinesin-12*-like (positioned within *PIK3C2A*, on the opposing strand; supplementary table S4, Supplementary Material online). Benowitz et al. (2022) identified a premature stop codon in *kinesin-12*-*like* (hereafter, *kinesin*-12) as the primary candidate target of selection underlying Cry1Ac resistance in the field-derived GA-R strain of *H. zea*.

Therefore, we investigated whether this same mutation occurred in our wild-caught samples. Requiring 95% confidence, we could call the presence or the absence of the premature stop codon in 100 of our 237 samples. Of these, 99 carried the susceptible-strain allele and 1 carried a nonsynonymous C>A transversion mutating glutamine to lysine. We also identified 9 nonsingleton SNPs in the coding region of *kinesin*-12 that were both nonsynonymous and resulted in amino acids with different biochemical properties to that of the susceptible strain (supplementary table S5, Supplementary Material online). The most common of these was a C>A transversion mutating threonine to lysine, which occurred at allele frequency q=0.11 (*N* = 96 genotyped individuals). Therefore, the premature stop codon is unlikely to have caused the putative sign of selection we observed, though it is plausible that other nonsynonymous mutations have a similar phenotypic effect in disrupting the function of Kinesin-related protein 12. It is also possible that unobserved indels, larger structural variants, or cis-regulatory elements could cause the putative signal of selection observed at this locus. We note that the highest CLR values in this region align with segments immediately flanking *kinesin-12* (Fig. 8).

## Discussion

### Gene Flow Within and Between *Helicoverpa* Species in North America

Our first aim was to test for the presence of invasive *H. armigera* ancestry in the North American *H. zea* population. Based on its rapid spread in South America, high propagule pressure, and availability of suitable contiguous habitat, Kriticos considered it was “a matter of time” until *H. armigera*—or introgressed *H. armigera* ancestry, at least—establishes in North America (Kriticos et al. 2015). Despite this, to our knowledge there has been no reported evidence of *H. armigera* in Central America. There has only been one report of *H. armigera* captured in the United States prior to 2019, based on the COI haplotype of three individuals captured in Florida in 2015, with no evidence of establishment (Tembrock et al. 2019). We present the first conclusive report of admixed individuals in mainland North America. Only two individuals we sampled were admixed, and both were sampled at the same site in Texas despite an absence of population structure. The admixed samples show nowhere near the extent of *H. armigera* allele sharing observed in Brazil (Fig. 2a). We saw no evidence of *H. armigera* introgression in previously reported samples collected in 2012 or 2017, though the sampling effort was far reduced in those years compared to our 2019 survey. Overall, the results point to a small degree of recent interspecific gene flow concentrated at a pesticide resistance locus.

Haplotypes break down over successive generations through recombination and mutation (Pool and Nielsen 2009). The size and therefore the detectability of introgressed ancestry blocks therefore also decays over time. Admixture between *H. armigera* and *H. zea* in South America was apparently punctuated by a pulse of hybridization 60 to 100 generations before 2017, with declining rates of hybridization since (Valencia-Montoya et al. 2020). We concluded that the vast majority of *H. zea* samples were nonadmixed. Since we do not expect ABBA and BABA pattern frequencies to be exactly equal, values of $f_{d}$ insignificantly greater than zero across several chromosomes (Fig. 1b) are consistent with an absence of introgression. This does not rule out the possibility of rare, short *H. armigera* haplotypes segregating in the *H. zea* population. However, our method was sensitive enough to detect *H. armigera* introgression in a region spanning ∼1% of the genome in <1% of individuals, suggesting that it was sufficient for our aim of identifying the presence of introgression at levels that are meaningful for the management of this pest.

Our second aim was to characterize population structure within nonadmixed North American *H. zea*.

While the use of genomic data has revealed otherwise cryptic population structure in some species, including *H. armigera* in its native range, we show that previous reports of panmixia in *H. zea* are not simply due to a lack of power (Zhang et al. 2022). Our analysis based on millions of SNPs showed that individuals collected at different locations in Texas were, on average, as genetically differentiated from one another as they were from individuals collected in North Carolina. While remarkable, this result is consistent with our understanding of *H. zea* movement ecology. In the United States, a high proportion of *H. zea* adults are known to emigrate long distances from where they eclose due to high climb-rates shortly after take-off and wind-assisted dispersal (Jones et al. 2018; Lawton et al. 2022). Given that summer incursions at latitudes higher than our sampling range are consistently repopulated by southern populations in the range we sampled, *H. zea* is likely panmictic across North America. This is not to suggest that there will be no spatial pattern during the early stages of a selective sweep, but it does imply that there will be little geographic resistance to the spread of an advantageous allele. This highlights the need for monitoring and management at the species level across the agroecosystems potentially affected by *Helicoverpa*. Moreover, the evolution of pesticide or Bt resistance in one crop or region has immediate consequences throughout North America, where there may be substantially different agricultural and pest management practices (Reisig and Kurtz 2018). This result highlights the intrinsic ecological relationship between management practices in neighboring fields, regions, or states, and should emphasize the need for multilateral management solutions.

### Introgression of *CYP337B3* From Invasive *Helicoverpa armigera*

*CYP337B3* could have been introduced via two routes: northward dispersal of admixed individuals from South America, or an independent introduction of *H. armigera* from its native range. An independent introduction is highly unlikely as it would require many generations of hybridization and recombination. Yet we saw no evidence of *H. armigera* ancestry in 2012 and 2017. Gene tree reconstruction showed that both admixed samples were assigned to the same subclade as all but one Brazilian *H. armigera* sample (supplementary fig. S6, Supplementary Material online). Bootstrap support within clade 1 was low due to short branch lengths, indicative of selection (supplementary fig. S7, Supplementary Material online). It is therefore likely that the admixed samples carry the *CYP337B3* allele that is under selection in Brazil, which had reached high frequencies in Brazilian *H. armigera* and *H. zea* by 2017 (Valencia-Montoya et al. 2020). The very fact that *H. armigera* ancestry is concentrated around the *CYP337B3* locus, which is overrepresented among admixed individuals in Brazil due to selection, strongly suggests that the polymorphism was introduced through northward dispersal.

Our results show that the admixed samples carried alleles that, in *H. armigera* at least, have a semidominant phenotypic effect enabling resistance to fenvalerate, deltamethrin, and possibly cypermethrin (Joußen et al. 2012; Rasool et al. 2014; Ni et al. 2023). Although functional work is required to demonstrate that *CYP337B3* confers the same phenotype in a *H. zea* genomic background, it has a similar phenotypic effect in multiple divergent *H. armigera* genomic backgrounds, and has clearly been subject to strong selection during adaptive introgression in Brazilian *H. zea* (Walsh et al. 2018; Valencia-Montoya et al. 2020). The introgression of this gene into North American *H. zea* is an indirect result of adaptation to pyrethroid exposure both in the native range of *H. armigera* (Joußen et al. 2012; Rasool et al. 2014; Ni et al. 2023) and subsequently in South American *H. zea* (Durigan et al. 2017; Valencia-Montoya et al. 2020). The presence of the allele in North American *H. zea* does not necessarily suggest that it is locally adaptive. However, fenvalerate, deltamethrin, and cypermethrin are widely used in the United States, at least as of 2018, much as they are in Brazil and parts of the *H. armigera* native range (National Agricultural Statistics Service 2019). Thus, an otherwise absent resistance phenotype was introduced to the North American *H. zea* population via interspecific introgression. Recent work following up from our results showed that the frequency of *CYP337B3* among *H. zea* collected in Colorado in 2023 increased to 0.15 (Nufer et al. 2024)—two orders of magnitude greater than the 2019 frequency we report here (0.0042 to 0.0048). Although the low allele frequency reported here means that the allele did not significantly contribute to field resistance phenotypes in 2019, the introduction of this allele created the conditions for the spread of resistance via a process far faster than de novo mutation.

As anthropogenic activity increasingly brings pest species into secondary contact, introgression could become an important mechanism of rapid adaptation. This is particularly relevant to pesticide resistance, which can in some cases be underlain by a modular genetic architecture. For example, adaptive introgression of a *vkorc1* allele from the western Mediterranean mouse *Mus spretus* into the house mouse *M. musculus domesticus* confers the latter with resistance to anticoagulant rodenticides (Song et al. 2011). Introgression of an *ARH* allele from Atlantic to Gulf killifish species enabled tolerance to anthropogenic pollutants, which was shown to be highly fitness-enhancing (Oziolor et al. 2019). Similarly, interspecific introgression in the fungal genus that causes Dutch elm disease, *Ophiostoma*, was associated with virulence (Hessenauer et al. 2020). In each of these cases, genetic variation already shaped toward a fitness optimum in one species was introduced to another at relatively high frequency—an adaptive process that can be substantially faster than selection on de novo mutations (Marques et al. 2019).

### Rapid Adaptation in Nonadmixed *H. zea*

Taylor et al. (2021) measured genetic differentiation in North American *H. zea* between 2002 and 2017, concluding that the change in frequency of a chromosome 13 haplotype was the result of selection. We have confirmed this using multiple lines of evidence. Derived alleles in the sweep region clearly confer a strong fitness advantage. Anthropogenic selective forces such as pesticide or *Bt* exposure could explain the strength and timing selection, though we cannot rule out other causes of selection. Two gene annotations lie within the sweep region: *carboxypeptidase Q*-like and the cytochrome P450 gene *CYP333B3*. *CPQ*-like is noteworthy because some carboxypeptidases bind Cry1Ac in *Helicoverpa armigera* (Da Silva et al. 2018) and are up-regulated upon Bt exposure in other lepidopteran pests (Yang et al. 2018; Van Munster et al. 2007), though to our knowledge this specific gene has never been functionally associated with Bt resistance. Selection at *CYP333B3* is a more parsimonious explanation for the sweep.

Cytochrome P450s have repeatedly been implicated in the evolution of resistance to a range of pesticides, especially in *Helicoverpa*, via xenobiotic metabolism (Nauen et al. 2022). Studies of noctuid pests have shown that *CYP333B3* is induced by a broad array of pesticide classes: indoxacarb (a voltage-dependent sodium channel blocker), fluralaner (a GABA-gated chloride channel allosteric modulator), imidacloprid (a nAChR competitive modulator), aldrin (an organochlorine), chlorantraniliprole (a ryanodine receptor modulator), several host plant defences (xanthotoxin and gossypol), and the pyrethroid fenvalerate (de la Paz Celorio-Mancera et al. 2011; Yangchun 2014; Wang et al. 2018; Amezian et al. 2021; Shi et al. 2022).

In terms of studies of *CYP333B3* in *Helicoverpa,* Yangchun et al. showed that *CYP333B3* is constitutively overexpressed in pyrethroid-resistant *H. armigera* (Yangchun 2014). *H. zea* resistance to pyrethroids was first reported in 1990, and control failures became apparent in the South and Mid-West in the mid-2000s when derived *CYP333B3* allele frequencies were approaching 0.4 (Jacobson et al. 2009). Pyrethroid resistance was found to be widespread in Texas in the mid-2000s (Pietrantonio et al. 2007). By the late 2010s, pyrethroids were considered to have mixed efficacy compared to other commonly used pesticides (Olmstead et al. 2016a, 2016b). This change in phenotype is consistent with the allele frequency trajectory we observe. Through the application of the P450 synergist piperonyl butoxide, multiple studies found that resistance to the pyrethroid cypermethrin was mediated by the activity of enzymes such as cytochrome P450s (Kanga et al. 1996; Li et al. 2000). In contrast to this result, Jacobson et al. (2009) performed a similar experiment in field-derived *H. zea* from Illinois, concluding that resistance to the pyrethroid bifenthrin was not P450-dependent. The genetic architecture of pyrethroid resistance is clearly complex and strain-dependent in *H. zea*. Although the timing of selection matches observed phenotypes in *H. zea*, the functional link between *CYP333B3* and pyrethroid resistance is not sufficiently clear to rule out other selective agents.

*CYP333B3* has a clearer functional association with organochloride resistance in *H. zea*. Shi et al. performed an in vitro metabolism assay in *H. armigera* and found that *CYP333B3* showed the highest activity for metabolism of the organochlorine aldrin (Shi et al. 2022). In the United States, Aldrin was banned in the 1980s along with other organochlorine pesticides, though in Mexico orgranochlorine use continued until 2000 (Alegria et al. 2005). Recent selection for resistance to legacy organochlorines is not completely implausible. For example, genomic monitoring combined with resistance assays of *Anopheles funestus* in Tanzania showed that recent selection at the *kdr* locus was driven by exposure to the banned organochlorine DDT, likely as a result of local pollution (Odero et al. 2024). Organochlorines such as Aldrin decay substantially slower in more temperate climates, with some half life estimates in the order of years to decades (Ghadiri et al. 1995; Bidleman and Leone 2004; Wong et al. 2008). There is some evidence that organochlorines have not decayed in agricultural soil tested throughout North America during the period 2003 to 2012 compared to the previous decade, possibly through occasional unlicenced use (Camenzuli et al. 2016). Organochlorines including Aldrin were detected at sufficiently high levels to suggest recent agricultural use, more than a decade after they were banned in Sonora, Mexico (Cantu-Soto et al. 2011). Even in the absence of unlicenced use, and even if such pesticides were below threshold levels measured for human safety, it is conceivable that *H. zea* pupating in soil would be exposed to legacy organochlorines including Aldrin. This is especially likely in Mexico where organochlorine use was more recent and intensive. Even so, we cannot rule out other selective forces affecting *CYP333B3* allele frequencies in the absence of a manipulative experiment.

In addition to its association with a wide variety of insecticides through xenobiotic metabolism, *CYP333B3* appears to play an important role in the development and metamorphosis of *H. armigera* (Zhang et al. 2016). The cause of the sweep at this locus could therefore reflect adaptation of a trait completely unrelated to insecticide resistance. Functional work is required to test the association between *CYP333B3* and insecticide resistance in *H. zea*.

Regardless of the specific pesticide(s) that imposed positive selection on derived *CYP333B3* alleles, it is clear that the adaptive response occurred within a 20-year time frame—an example of rapid anthropogenic adaptation. Although *CYP333B3* appears to be under convergent selection in *H. armigera* in Europe, Africa and South America (Jin et al. 2023), we see no evidence of introgression into *H. zea* from *H. armigera* at this locus. Rather, the increased rate of coalescence and genetic differentiation from *H. armigera* and at this locus, combined with the recorded increase in frequency from a rare variant in *H. zea* over a decade before the first observation of *H. armigera* in South America, strongly supports selection from de novo mutation within *H. zea*. Thus, while de novo adaptation may be slower than adaptive introgression, it can occur at timescales once thought to be impossible for an evolutionary process. Therefore, for species with large and highly connected populations exposed to strong selection pressures, management strategies must not only consider ecological impacts but also evolutionary change over the short- and medium-term.

We estimated that *CYP333B3* was subject to a selection of strength $\hat{s}\approx 0.05$, and showed that this coefficient could plausibly explain independently observed changes in the frequency of nonsynonymous mutations from 0.07 in 1998 to 0.87 in 2017 (Taylor et al. 2021; Fig. 7, supplementary table S6, Supplementary Material online). However, the selection coefficient estimate based on a fit to the observed data was slightly higher than the estimate obtained from the sweep. While the 2019 allele frequency estimate cannot be compared to allele frequency trajectories from other years due to methodological differences, the empirical estimate of the allele frequency in 2019 (0.956) was noticeably greater than the upper estimate modeled assuming $\hat{s}$ (0.88). There are many explanations for this difference toward the end of the time-series—and we note that the two estimates differed by a much smaller degree over most of the time-series—though it could be that $\hat{s}$ is conservative estimate of the strength of selection. Given that selection coefficients >0.01 are often described as “strong” (Bersaglieri et al. 2004; Zhao et al. 2023), the derived *CYP333B3* allele is clearly associated with a remarkable fitness advantage.

### Other Signatures of Selection in North American *H. zea*

Bt resistance in *H. zea* is a major concern. Extensive research has shown that resistance to some toxins (e.g. Cry1) is already common, and that selection for resistance to Cry2Ab2 was ongoing at the time we collected samples (Ali et al. 2006; Dively et al. 2016; Reisig et al. 2018; Bilbo et al. 2019; Yu et al. 2021). Although phenotypic data clearly show that resistance is evolving, identifying its genetic basis in both the field and the lab has been challenging. This is because the same phenotype can result from many polygenic, semi-overlapping genetic architectures. Multiple recent studies have identified Bt resistance QTL that do not overlap with any established Bt resistance loci (Taylor et al. 2021; Benowitz et al. 2022). Detecting the signature of selection on such complex traits is difficult without extensive replication or time-series data. Taylor et al. (2021) identified several QTL associated with Bt resistance but found no evidence of allele frequency shifts at these loci over time. It is perhaps not surprising, then, to find that none of the 19 Bt resistance QTL we investigated stood out as showing clear selective sweeps—especially since the approach we use is designed to detect hard selective sweeps, which are underpowered for detecting minor-effect QTL contributing to quantitative traits. One notable exception is the locus *PIK3C2A*/*kinesin-12* (Fig. 8), in the upper first percentile of CLR values genome-wide. Benowitz et al. (2022) identified a premature stop codon in *kinesin-12* as the primary Bt resistance candidate in their QTL. We did not observe this mutation in our field-collected samples, though we did see nonsingleton, nonsynonymous mutations in the coding region of *kinesin*-12 that altered the physio-chemical properties of amino acids and may have had the same phenotypic effect on disabling protein function. Cis-regulatory mutations could also disable the function of Kinesin-related protein 12; consistent with this hypothesis, the highest CLR values abut *kinesin*-12. So while the specific mutation observed by Benowitz et al. likely does not explain the putative signature of selection we observe, it is plausible that there has been selection at this locus in the field. Two important caveats should be noted. First, we could confidently call genotypes in ∼40% of our samples, so we cannot rule out selection on the premature stop codon itself in the field. Second, this locus is only noteworthy because of our specific candidate gene search; it would not have been included amongst the most likely targets of interest in our selective sweep analysis alone. Even though it is within the upper first percentile of CLR outliers, CLR values are distributed such that hundreds of other genes show more obvious signatures of selection (supplementary fig. S10, Supplementary Material online). Therefore, functional work is needed to test Benowitz's hypothesis that a premature stop codon in *kinesin-12*-like contributes to Bt resistance in the field.

To define sweeps in our hypothesis-free selection scan, we used a stringent yet arbitrary (upper 0.01st percentile of CLR values) in order to avoid false positives, though this means that real selective sweeps may be missed. For example, chromosomes 10 and 26 showed CLR values only 4 units below the threshold (supplementary fig. S10, Supplementary Material online). For two of the three putative sweeps we identified, we can only speculate on possible causes of selection. The sweep on chromosome 15 includes a *takeout*-like gene. *Takeout*-like genes are over-expressed in pyrethroid-resistant mosquitos and aphids; in the latter RNAi experiments showed that they directly contribute to resistance (Toé et al. 2015; Peng et al. 2021). *Takeout-*like genes were also significantly over-expressed in *Spodoptera litura* upon exposure to the isoxazoline insecticide fluralaner, and in honeybees exposed to the herbicide atrazine (Jia et al. 2020; Wang et al. 2023). It is therefore possible that this sweep is due to selection on a cis-regulator of *takeout*-like, though showing this requires further investigation. It is also noteworthy that the chromosome 15 sweep overlaps with two genes encoding E3 ubiquitin-protein ligases involved in protein degradation and cell cycle regulation (*Hyd-*like and *RNF168*-like) (Flack et al. 2017). Both *Hyd* and *RNF68* have been implicated in host-virus protein–protein interactions, consistent with selection imposed by a viral outbreak (Lilley et al. 2010). Some of the clearest signatures of selection result from outbreaks of infectious disease (Obbard et al. 2011). This may be particularly likely in North American *H. zea*, where nucleopolyhedroviruses are often used as a means of biocontrol alongside Bt toxins and synthetic pesticides (Niedermann et al. 2017).

The cause of the putative sweep on chromosome 25 is less clear, though we note that the dynein gene in this region was found to be the most up-regulated gene in an RNA-seq comparison of neonicotinoid-susceptible versus resistant honeybees (Bahia 2021). While there is insufficient a priori information to go beyond a simple description of the sweeps on chromosomes 15 and 25, the plausible cause of selection on chromosome 13 is more obvious.

### Pleiotropy, Epistasis, and the Evolutionary Fate of *CYP337B3* and *CYP333B3* Alleles

We identified two cytochrome P450 alleles recently introduced to the *H. zea* metapopulation via completely distinct evolutionary processes. If both are associated with pyrethroid resistance, this begs the question: is there an epistatic interaction between *CYP333B3* and *CYP337B3*, and if so, what does this mean for the future spread of *H. armigera* ancestry? If the derived allele of *CYP333B3* is common and *CYP337B3* is rare, is the marginal fitness of the latter completely diluted in this population? The relationship between *CYP337B3* and pyrethroid resistance is clearly not always straightforward, as evidenced by a lack of correlation between *CYP337B3* frequency and fenvalerate resistance in *H. armigera* collected in China due to the activity of other cytochrome P450s (Han et al. 2015). Several factors need to be considered. First, resistance to a specific pesticide is a quantitative trait for which distinct cytochrome P450 genes may have additive effects (Brun-Barale et al. 2010; Tchouakui et al. 2021). Second, pyrethroids represent a vast and diverse set of pesticides. No single mutation confers complete resistance to all pyrethroids, and different mutations associated with pyrethroid resistance can contribute to resistance in a nonoverlapping set of pesticides (Sparks et al. 2012; Wang et al. 2021). In other words, both resistance alleles are likely to have some degree of pleiotropic phenotypic effect. Third, the derived *CYP333B3* allele was already common in North America when *CYP337B3* was introduced to South American *H. zea* populations in 2013 (Fig. 7b). *H. zea* populations are well connected between North and South America, as evidenced by the spillover of *H. armigera* ancestry that we report here. Pyrethroids have been commonly used in both Brazil and the United States for pest control in cotton and maize (Walsh et al. 2022). Given that *CYP337B3* was highly fitness-enhancing in South America, and given that it would likely have often occurred in a genomic background with this same *CYP333B3* allele, we have little reason to think it would not spread in North America as well. Recent work suggests the allele has spread significantly since 2019, though this could result from additional gene flow, selection, or a combination of both (Nufer et al. 2024).This raises a fourth factor to consider: demography. Due in part to the phase of the El Niño southern oscillation, a population expansion of *H. armigera* in Brazil shortly after its detection appears to have resulted in demographic swamping, producing a pulse of hybridization (Valencia-Montoya et al. 2020; Specht et al. 2021). By contrast, in 2019 we see a “trickle” of *H. armigera* ancestry into North American *H. zea*. Even alleles under strong selection can be lost through drift at sufficiently low frequency. Therefore, the ongoing influx of *H. armigera* ancestry and the North American selective regime with respect to *CYP337B3* will together determine spread. While a degree of predictive power could be gained through fitness assays to determine the epistatic interaction between *CYP337B3* and *CYP333B3* for tolerance to fenvalerate and other commonly used pesticides, the outcome of this incursion remains to be seen through future biosurveillance.

### Prospects for Genomic Surveillance in This System and Others

Early detection of invasive pests can pay dividends even when surveillance is costly, as invasive species are easier to manage shortly after they establish (Mehta et al. 2007). Genomic detection of *H. armigera* is currently far more scalable and sensitive as a diagnostic test to distinguish *H. zea* from *H. armigera* relative to morphological or single-locus genetic assays. Nonetheless, a trade-off exists between the cost of surveillance and the product of the probability and cost of invasion. Given that *H. armigera* ancestry has spread into the United States, and the value of the crops exposed to *H. armigera* (∼US$78 billion per annum; Kriticos et al. 2015), there is a strong case for extensive ongoing genomic monitoring of *H. zea* across North America, especially in the dispersal corridor leading through Central America and Mexico where there has been comparably little genomic monitoring to our knowledge. Data collected over a time series are particularly powerful for studying invasive species (Kim et al. 2023). Previously collected tissue samples from Central and North America will be valuable sources of information for tracing the spread of *H. armigera* and alleles of interest. However, this relies on the dependable stewardship of publicly available genomic data and associated spatiotemporal metadata (Toczydlowski et al. 2021; McGaughran et al. 2024). The publicly available samples used in this study are a testament to this.

Monitoring programs must also adopt a genic view of biological invasion in order to quantify the incursion of *H. armigera* (North et al. 2021). This means that, if the aim is to map the geographic distribution of possible *H. armigera* spread, *CYP337B3* assays are likely to be more informative than ancestry-based assays that use one or few markers. Alternatively, if the aim is to determine the presence of *H. armigera* introgression in an area of concern, only genomic data offer the resolution required to detect the short haplotypes segregating in the population. Future work to characterize the sets of loci most sensitive to admixture (e.g. those in high-recombining regions, least likely to be removed by selection due to linkage with Bateson–Dobzhansky–Muller incompatibilities) will allow for the development of SNP arrays that can robustly assign ancestry proportions to samples at lower cost.

When genomic data were first adopted in the field of molecular population genetics, there was excitement about the power of population genomics to infer the evolutionary history and genetic basis of adaptive traits in wild populations, especially where lab crosses were impossible (Li et al. 2008). While genomic surveillance has become a critical technology for the management of pests and invasive species (Chown et al. 2015; Hamelin and Roe 2020), in practice the detection of adaptation can be limited by the complex genetic architectures and demographic nonequilibrium. Incorporating QTL metadata and sampling over a time-series can address these issues—reinforcing the need for sustained genomic monitoring programmes—though this is not always possible (Pélissié et al. 2018; Taylor et al. 2021; Clark et al. 2023). Although we only sample individuals at a single timepoint, we show that relevant population genetic statistics can be estimated with sufficient accuracy to not only identify a putative target of selection for pesticide resistance, but to precisely infer recent allele frequency change at the locus. The analyses we apply here demonstrate the richness of information that can be extracted from nucleotide diversity in wild pest populations to inform management action and study anthropogenic adaptation.

In summary, we have shown that fitness-enhancing genetic variants were introduced to North American *H. zea* recently, and rapidly, via two independent processes: interspecific introgression and intraspecific adaptation. These findings underscore the importance of rapid adaptation for pest management—evolution at timescales previously only considered to be relevant for ecological process.

## Materials and Methods

### Sampling, DNA Extraction, Library Preparation and Sequencing

*Helicoverpa zea* individuals were collected in agricultural fields at 10 locations across the southern United States in 2019 (see supplementary table S1, Supplementary Material online and Fig. 1) using corn earworm pheromone lure (GreatLakes IPM, Vestaburg, MI). Adult moths were immediately placed in 95% ethanol and stored at −20 °C prior to DNA extraction. A few populations of *H. zea* were collected as larvae (see supplementary table S1, Supplementary Material online) and brought back to the laboratory of Dr. David Kerns at the Texas A&M University Dept. of Entomology in College Station, TX, USA where they were raised to adults and then processed for DNA extractions.

The abdomen of each adult moth was separated from the body and used for DNA extraction with the Qiagen DNeasy Blood & Tissue kit (Qiagen, Germantown, MD following the manufacture's protocol. The final DNA elution step was performed using Qiagen buffer EB (Qiagen, Germantown, MD) instead of the AE buffer. DNA concentration was measured using a Qubit 3.0 Fluorometer (Thermo Fisher Scientific, Waltham, MA). Genomic DNA samples were delivered to the Texas A&M AgriLife Genomics and Bioinformatics Service in College Station, TX for library preparation and sequencing.

DNA batch purity and integrity were assessed using a DS-11 spectrophotometer (Denovix, Wilmington, DE) and capillary gel electrophoresis (Fragment Analyzer, formerly Advanced Analytical, now Agilent, Santa Clara, CA), respectively, from 11 samples per 96-well plate. Genomic DNA was further purified with SPRI beads (Omega Bio-tek, Norcross, GA) and the concentration of each sample was determined using Lunatic plates (Unchained Labs, Pleasanton, CA) read on a DropletQuant spectrophotometer (PerkinElmer, Waltham, MA).

Libraries were prepared from 33 ng of genomic DNA using a custom-miniaturized version of the NEXTFLEX Rapid XP kit protocol (PerkinElmer) that was automated on a Sciclone NGSx liquid handler (PerkinElmer). Briefly, all reactions involving enzymes were carried out in one-third of manufacturer proscribed volumes for each reagent. In the modified reaction volumes, genomic DNA was enzymatically fragmented for 3 min, then ligated to unique dual-indexed barcodes. The raw library reaction was brought up to protocol volumes with H_2_O for a SPRI cleanup followed by SPRI size selection between 520 and 720 bp, and then eluted in reduced volume to be amplified for 10 PCR cycles. Finally, the amplification reaction was brought back up to recommended volume with H_2_O to perform an additional single-sided SPRI size selection that retains DNA fragments larger than 450 bp.

A subset of libraries was checked for size and integrity on the Fragment Analyzer and all libraries were quantified using a fluorescent plate reader (SpectraMax M2, Molecular Devices, San Jose, CA) with PicoGreen reagent as per the manufactures suggested protocol (Thermo Fisher Scientific, Waltham, MA). Libraries were diluted with EB (Omega Bio-tek) to a final concentration of 2.25 ng/µl using an automated liquid handler (Janus, PerkinElmer) and an equal volume of each was pooled. Pooled library quality was assessed on the Fragment Analyzer and molarity determined using a qPCR-based Library Quantification assay (Roche, Pleasanton, CA).

The pool was sequenced in a single lane of an Illumina NovaSeq S4 XP flowcell (San Diego, CA) using the 2× 150 bp recipe. The raw data were demultiplexed with bcl2fastq 2.20, which yielded 2.17 billion demultiplexed reads ranging from 5.01 to 10.41 M reads per sample and an average of 7.80 M reads per sample (∼6.5× coverage).

### Filtering, Alignment, and Variant Calling

In order to conduct intra- and inter-specific comparisons, we used raw sequencing data from multiple *Helicoverpa* spp. generated by Anderson et al. (2018), Taylor et al. (2021) and Jin et al. (2023). These publicly available data were analysed in the same manner as our newly generated sequence data. Two slightly different pipelines were used for different analyses depending on the samples required. Bioinformatics pipelines were implemented using Snakemake v7.2 (Mölder et al. 2021). All samples were mapped to the *Helicoverpa armigera* reference genome (Pearce et al. 2017) to maximize sensitivity when detecting introgression from *H. armigera*, and to allow direct comparison with results generated in similar studies (Valencia-Montoya et al. 2020; Taylor et al. 2021).

For the results presented in Figs. 1 to 5 (hereafter, Call Set 1): Fastq files were trimmed using fastp v0.23 (Chen et al. 2018), then mapped using bwa v 0.7.12 (Li and Durbin 2009). The mean percentage of reads mapped (after QC filtering) was 74.36% across all newly generated samples collected in 2019. The resulting .bam files were sorted using SAMtools (Li et al. 2009). Picard v2.9.2 (Broad Institute 2023) was used to remove duplicates and SAMtools was used to index the filtered .bam files. GATK HaplotypeCaller v4.3 (Van der Auwera et al. 2013) was used to call haplotypes per individual. GATK CombineVCFs was used to merge call sets across all individuals and GenotypeGVCFs was used to jointly call genotypes across samples sequenced here, those produced by Anderson et al. and samples collected in 2002 by Taylor et al. (2021). Of these, only single nucleotide polymorphisms were retained. VCFtools (Danecek et al. 2011) was used to filter out sites with a phred-scaled quality score below 20, sites with a mean depth of coverage below 2× or above 200×, genotypes with a mean depth below 2× or above 200×, with a missing data threshold of 50%.

Although the use of the *H. armigera* reference assembly was crucial for our aims, the choice of reference genome can influence mapping rates and allelic bias if there is sufficient genetic divergence between the reference and the samples. We confirmed that our samples mapped at similar rates for the *H. armigera* reference assembly (74.36%) compared to the *H. zea* assembly (Benowitz et al. 2022; 85.6%). The MQRankSum test statistic calculated using GATK was used to confirm that there were no signs of allelic bias/dropout. Before filtering, 8 SNPs of 46,200,314 potentially heterozygous sites show MQRankSum values >20 or <20, and one such SNP remained after filtering (HaChr29: 2685436).

The *H. armigera* genome is highly contiguous with the *H.* zea chromosome except for the sex chromosome (Benowitz et al. 2022). Therefore, some *H. zea* sex chromosome or pseudo-autosomal reads miss-mapped to autosomal scaffolds in the reference assembly. To avoid potential biases introduced through miss-mapping, Plink v1.9 (Chang et al. 2015) was used to calculate the statistical association between each autosomal SNP and heterozygosity on the Z chromosome, in which Z chromosome heterozygosity was treated as a quantitative trait. SNPs with *P* values in the top first percentile of association were excluded from all downstream analyses. This resulted in a call set of 45,700,390 SNPs among 304 individuals of four species (supplementary table S2, Supplementary Material online). For analyses requiring additional samples (e.g. *H. zea* from 2017 and 2012, and those shown in supplementary fig. S4 to S7, Supplementary Material online), mapping and variant calling was completed using a subset of relevant samples jointly called using the same pipeline.

For the selection analysis (results presented in Figs. 7 and 8; hereafter Call Set 2), a more stringent filtering pipeline was used. Paired end raw reads were trimmed using Trimmomatic v0.38 (parameters: LEADING:3 TRAILING:3 SLIDINGWINDOW:4:15 MINLEN:50) (Bolger et al. 2014). Trimmed and paired reads were aligned to the *H. armigera* reference assembly generated by Pearce et al. (2017). Alignment was performed using bwa (Li and Durbin 2009) and mate pairs were fixed using SAMtools v1.9 (Li et al. 2009). Resulting bam files were sorted using SAMtools. Picard v2.18 was used to add read groups, clean bam files, and mark duplicates. Subsequently, SAMtools was used to index the bam files, and GATK HaplotypeCaller v4.15 (Van der Auwera et al. 2013) was used to call haplotypes individually for each sample. Haplotypes were imported into a database using GenomicsDBImport from GATK. Lastly, GATK's GenotypeGVCFs was used to call SNPs from all samples jointly. Next, the called genotype in a vcf file was subjected to a sequential filtering process using VCFtools v0.1.16 (Danecek et al. 2011). First, we removed samples that had over 50% of loci missing; these samples usually resulted from poor sequencing coverage. Second, we applied a quality filter by removing loci that had a genotype quality score lower than 20 (minGQ 20). Third, we applied a depth filter to remove loci that had coverage less than 2 or greater than 200. We only kept biallelic SNPs and removed all indels. Next, we removed loci missing from more than 50% of the samples missing, as well as all singletons in the vcf file. In the next step, we filtered out loci that violated Hardy–Weinberg equilibrium (HWE). HWE filtering was only conducted for *H. armigera* and *H. zea* samples because of the much smaller sample size of other species. HWE tests were applied only to loci with no missing genotypes, and *P*-value cutoff was set to 0.01. Loci that violated HWE in two of the three species were removed. We imputed the missing genotypes using Beagle (v5.1, Browning et al.) with default settings. Then we only kept biallelic SNPs in the imputed genotypes, and we also applied linkage disequilibrium pruning using Plink (−indep-pairwise 50 10 0.5). SNPs associated with Z chromosome heterozygosity were filtered using the method described above, resulting in a set of 5,706,238 SNPs across 237 *H. zea* individuals.

For most window-based analyses described below, a window size of 20 kbp was chosen because linkage disequilibrium decayed to a genome-wide background level of $r^{2}$ = 0.002 over approximately 20 kbp for the population of North American *H. zea* that we sampled.

### Detecting Introgression

To test for allele sharing with South American *H. armigera,* we calculated $\hat{\;f_{d}}$ in 20 kbp windows across all chromosomes in which at least 200 SNPs were called, using Python scripts described in Martin et al. (2015). P1 was designated as a set of 13 *H. zea* individuals sampled in 2002 in Louisiana by Taylor et al. (2021). These samples were collected over a decade before *H. armigera* was detected in the Americas and therefore represent the best possible whole-genome reference set of nonadmixed *H. zea*. P3 was 25 nonadmixed *H. armigera* sampled in Brazil, and the outgroup was 7 *H. punctigera* individuals sampled in Australia. For our samples, $\hat{\;f_{d}}$ was calculated 10 times where the test set, P2, was *H. zea* sampled in 2019 at each of the 10 samples sites. An additional three tests were carried out using *H. zea* collected by Taylor et al. (2021) in 2012 in Louisiana, in 2017 in Maryland, and in 2017 in Louisiana. As a positive control, $\hat{\;f_{d}}$ was also calculated for 9 individuals sampled in Brazil shown to be admixed offspring of *H. armigera* and *H. zea* by Anderson et al. (2018). Results from all 14 tests are shown in Fig. 1.

### Calculation of Summary Statistics and Visualization With PCA

To compare the two admixed individuals with *H. armigera* samples, we calculated nucleotide diversity (*π*), genetic differentiation ($F_{ST}$), and genetic divergence ($d_{xy}$) for the results presented in Fig. 3 and supplementary fig. S2, Supplementary Material online using python scripts described by Martin et al. (2015). This was done in 20 kbp and 100 kbp windows (with at least 200 and 100 informative sites, respectively) with consistent results. Principal components analysis (Fig. 4) was carried out using Plink v1.9 (Chang et al. 2015) in segments A and B separately. We also conducted principal components analysis across all autosomes using the same method (supplementary fig. S8, Supplementary Material online).

### Reconstructing Gene Trees

To reconstruct a tree at the *CYP337B3* locus, we used a combination of *H. zea* samples collected here (two admixed and two representative nonadmixed samples), 4 *H. zea* collected in 2002 by Taylor et al. (2021), 2 *H. punctigera* and 9 *H. armigera* collected from the invasive range in Brazil by Anderson et al. (2018) (which are admixed with *H. zea*), and 10 *H. armigera* from the major clades of the native range by Jin et al. (2023) (see supplementary table S2, Supplementary Material online). These samples were mapped, jointly genotyped and filtered without phasing in the manner as described above. We used VCFtools v0.1.15 (Danecek et al. 2011) to subset the *CYP337B3* locus previously used to identify signatures of adaptive introgression (HaChr15:11436565-11440168; Valencia-Montoya et al. 2020) from a multigenome VCF and converted to PHYLP format. We removed invariant and uninformative sites and retained sequences with no more than 50% missing data. We reconstructed a maximum-likelihood phylogram the GTR + GAMMA model of rate heterogeneity and Lewis ascertainment bias correction method implemented in RAxML v 8.2.12 (Stamatakis 2014) with 100 bootstrap iterations. We rooted the tree using the two outgroup *H. punctigera* samples in ggtree (Xu et al. 2022), ensuring bootstrap values were assigned to the correct node with the edgelables function (Czech et al. 2017). The resulting cladogram is shown in supplementary fig. S4, Supplementary Material online. The bootstrap values, tip label sample names and scaled branch lengths are shown as a phylogram in supplementary fig. S5, Supplementary Material online, which was rooted in FigTree v1.4.4.

Since bootstrap support was low within the *H. armigera* clade but high for nodes separating species clades (supplementary fig. S4 to S5, Supplementary Material online) we repeated the tree reconstruction using the same method with a constrained multifurcating topology [((*armigera*, *zea*), *punctigera*)]. The constrained topology tree, show in supplementary figs. S6 and S7, Supplementary Material online, improved the likelihood (−455.580095 compared to −455.580278).

### Quantifying Isolation by Distance and Population Structure

To investigate connectivity across the North American *H. zea* metapopulation, we calculated the mean of $F_{ST}$ across 20 kbp windows from all chromosomes for each of 45 possible pairwise combinations of the 10 sampling locations. Geographic distance was calculated for the same pairwise combinations using the R package geosphere (Karney 2013). We regressed $\frac{\bar{F_{ST}}}{\left(1-\bar{F_{ST}}\right)}$ on log-transformed geographic distance to test for a positive correlation. A positive correlation would suggest a pattern of isolation-by-distance and provide a rough estimate of the product of population density and the variance in dispersal distance, whereas the absence of a correlation would indicate panmixia across sampling locations (Rousset 1997).

To further investigate population structure, we used *structure threader* to implement *fastStructure* (Raj et al. 2014; Pina-Martins et al. 2017). We used autosomal sites from call set 1 with a minor allele frequency (MAF) of at least 0.05 within 20 kbp of one another. We investigated values of *K* < 6 (supplementary fig. S9, Supplementary Material online) and found the same results with and without the MAF filter.

### Identifying Selective Sweeps

Briefly, we identified selective sweeps as localized and extreme deviations from the genome-wide site-frequency spectrum (SFS) consistent with linked selection using the CLR test implemented in *SweepFinder2* (Nielsen et al. 2005; DeGiorgio et al. 2016). To do so, we used sequence data from other *Helicoverpa* species to distinguish ancestral from derived alleles. By using the empirical SFS as a null model, rather than an equilibrium null SFS, we account for the confounding influence ofemographic nonequilibrium (Nielsen et al. 2005). We use sequence data from other *Helicoverpa* species to estimate chromosome-wide mutation rates in order to estimate the population recombination parameter, then use chromosome-wide recombination rate estimates and estimates of the effective population size to estimate the strength of selection that acted at a locus of interest. Finally, we show that the estimated selection coefficient can explain independently observed shifts in allele frequency.

Specifically, we used VCFtools v0.1.15 (Danecek et al. 2011) to calculate allele frequencies within the North American *H. zea* population sampled in 2019. Including 25 nonadmixed *H. armigera* individuals and 7 *H. punctigera* individuals as outgroups, we used a custom python script to designate ancestral and derived states to those alleles and calculate all possible transition and transversion rates for each chromosome. With these ancestral and derived states, *SweepFinder2* (DeGiorgio et al. 2016) was used to compute the unfolded genome-wide site frequency spectrum in the North American *H. zea* population, and to use this spectrum as a null model in a scan for selective sweeps. Briefly, *SweepFinder2* runs a CLR test to identify chromosomal regions that show a decline in genetic diversity characteristic of genetic hitchhiking (Kim and Stephan 2000). The CLR was computed in 1 kbp intervals. Note that the LD-based local recombination rate estimates generated below were not used to detect the location of selective sweeps, as this would have confounded the localized effect of selection on both the site-frequency spectrum and on linkage disequilibrium. We identified sites of interest (those clearly showing signs of selection) as the top 0.01st percentile of CLR values (CLR > 104.17). Sites above this threshold within 20 kbp of one another were grouped into clusters using BEDtools v2.20.1 (Quinlan and Hall 2010), whereby the cluster start and end positions extended 20 kbp up/downstream of the terminal outlier sites. We refer to these regions as putative selective sweeps.

### Mapping Known Candidate Bt Resistance Loci

We identified three classes of previously identified candidate Bt-resistance loci: genes within the QTL described by Benowitz et al. (2022); genes repeatedly implicated in Bt resistance which were also examined by Taylor et al. (2021); and the novel resistance QTL identified by Taylor et al. (2021).

First, Benowitz et al. (2022) mapped a 250 kbp Bt resistance QTL to chromosome 13 containing 10 genes of interest, including the putative causative locus *kinesin-12*-like, using a different reference assembly. To test for signatures of selection in this region, we used BLAST v2.4 to map each of the 10 loci in Table 2 of Benotitz et al. to our reference assembly, retaining the largest region of overlap with an *E*-value of 0 for each locus. As expected given the contiguity between our assemblies, all loci mapped within 250 kbp of one another on chromosome 13. Within each BLAST hit, we used BEDtools v2.20.1 BEDops 2.4.41 to identify annotations in our assembly with matching annotation names (Quinlan and Hall 2010; Neph et al. 2012). Two annotations (*kinesin-12*-like, *JHE*-like) were absent in our annotation; we excluded the latter because we could not confirm its location. Because *kinesin-12*-like mapped well to our assembly (>90% sequence identity, *E*-value = 0), and since *kinesin-12*-like sits within the larger gene *PIK3C2A* (where it is encoded on the opposite-sense strand) we could map the 1.7 kbp gene with a range of error <50 bp.

Second, we applied the same mapping approach to identify other known Bt-resistance loci listed in Taylor et al. (2021)  supplementary table S4, Supplementary Material online. We were able to confidently assign corresponding annotations for all genes except alp, cad2, *calp4,* and *abcA2*.

Third, Taylor et al. (2021) identified many QTL associated with resistance to crops expressing both Cry1Ab and Cry1A.105 plus Cry2Ab2 toxins. Both traits were highly polygenic. We identified the most significant outlier scaffold for both traits and mapped these to our assembly. This was done for separately for *H. zea* scaffolds and *H. armigera* super-scaffolds. To be consistent with Taylor et al. as much as possible, we defined the most significant outlier scaffold as that with the lowest mean *P* value for the linear mixed model likelihood ratio test with at least one SNP assigned a BSLMM posterior inclusion probability >0.01. We retained only super-scaffolds with at least 10 SNPs and scaffolds with at least 3 SNPs. We identified the scaffold KZ118765 (nested within NW_018395566) as the scaffolds most associated with Cry1Ab resistance. NW_018395399 and the nested KZ118015 were most associated with Cry1A.105 + Cry2Ab2 resistance. All four were within the resistance-associated linkage group 9 in the map produced by Taylor et al. and neither were associated with growth rate on either of the control treatments. We mapped these scaffolds to our reference assembly, retaining the largest hit with an *E*-value of 0 as above.

For all candidate Bt-resistance loci that we mapped, we performed a permutation test to determine the probability that the locus overlapped with outlier sweep CLR values by chance. To do so, we randomized the position of a locus of the same size in order to generate a null distribution of overlapping mean and maximum CLR values (supplementary table S4, Supplementary Material online).

### Calling *Kinesin-12* Genotypes

To directly compare *kinesin*-12 genotypes to those reported by Benowitz et al. (2022), we mapped reads to the full *kinesin*-12 sequence (including untranslated regions and introns) of the LAB-S (laboratory susceptible) *H. zea* strain. This was done to rule out the potential effect of alignment errors on genotype calls and enable an unbiased comparison of genotypes with our wild-caught individuals. This is because the *H. armigera kinesin*-12 sequence differs structurally from that of *H. zea* in noncoding regions, though its position in the genome is concordant. For all 237 2019 samples, the same pipeline applied to call set 1 was used to process fastq files, map reads and call variants with HaplotypeCaller. Three different parameters were used: the LAB-S *kinesin*-12 sequence was used as the reference sequence for mapping, no more than two alternate alleles were permitted per site, and monomorphic sites were retained. When calling variants, a cut-off of 95% confidence was used (reference genotype quality or QUAL > 13.0103 for monomorphic and polymorphic sites, respectively). Putative indels were not considered. The resulting sequences were translated using EMBOSS Transeq and aligned (along with the coding sequence of the Bt-resistant strain) using Mview (Madeira et al. 2022). This alignment confirmed that the Bt-resistant line carried at C>T mutation resulting in a premature stop codon, and allowed us to directly compare our samples.

We characterized nonsynonymous mutations of interest that could be confidently called in our samples. We reasoned that amino acid changes with novel biochemical properties are more likely to impact enzymatic function, that singleton mutations are more likely to be erroneous genotype calls, and that singleton mutations are less likely to occur at sufficient frequency to cause the putative signature of selection in the region. Therefore, we only report nonsynonymous mutations (relative to the coding region of the reference LAB-S complete sequence) that (1) produce amino acids with different biochemical properties, and (2) could be confidently called in more than one individual. Only single nucleotide polymorphisms (homozygous and heterozygous) were reported (supplementary table S5, Supplementary Material online).

### Estimating the Selection Coefficient, s

For each site in which the CLR was calculated above, *SweepFinder2* was also used to calculate Durrett and Schweinsberg’s (2004) approximation of *α*, where


$$s=\;\frac{r.\ln\;(2N_{e})}{\alpha }$$

This relationship allowed us to generate an estimate of the selection coefficient $\hat{s}$ in the sweep region on chromosome 13. To do so, we estimated the effective population size of our North American *H. zea* samples as $N_{e}$ = $\frac{{\hat{\theta }}_{\pi }}{4\mu }=$ 10.3 × 10^5^, using the *Drosophila melanogaster* mutation rate *μ* = 8.4 × 10^−8^ (Haag-Liautard et al. 2007) and ${\hat{\theta }}_{\pi }=\;$0.0346 (SD: 0.0252), calculated as the mean nucleotide diversity in 10 kbp windows using pixy (Korunes and Samuk 2021). This effective population size is approximately half that of native *H. armigera* populations and consistent with previous estimates (Anderson et al. 2018). We used the mean estimate of per-base pair recombination rate for chromosome 13 (see below): r=5.447 × 10^−8^. The lower and upper bounds of the selection coefficient estimate within the bounds of the *CYP333B3* locus (0.0446 < $\hat{s}$ < 0.0508) were calculated using 1 SD above and below the mean estimate of ${\hat{\theta }}_{\pi }$ (resulting in the upper and lower bounds of $N_{e}$ as 9.9 × 10^5^ and 10.6 × 10^5^, respectively). The mean estimate of the coefficient for the *CYP333B3* locus was $\hat{s}=$ 0.0489.

### Estimating Chromosome-Wide Recombination Rates

Estimation of the selection pressure, above, required mean per-chromosome estimates of the recombination rate. We estimated recombination rates across all chromosomes using patterns of linkage disequilibrium. First, we statistically phased data across all the *H. zea* individuals we sampled for all chromosomes using Beagle v5.0 (Browning and Browning 2007, 2016). We used 50 individuals with the highest depth of coverage to estimate the population recombination parameter *ρ* across all autosomes with LDhelmet v1.9 (Chan et al. 2012). We generated likelihood lookup tables using the same theta estimator described above, with the grid of *ρ* values between 0 and 10 specified with the command-r 0.0 0.5 3.0 1.0 10.0. Following the recommendation of Chan et al. (2012), 11 Padé coefficients were computed and, using a default block penalty of 50 and a window size of 50 SNPs, the Markov Chain Monte Carlo procedure was implemented for 10^6^ iterations with a burn-in of 10^5^ iterations. To compare observations with other data and confirm that chromosome-wide estimates of recombination rate were biologically realistic, we converted mean estimates of $\rho =4N_{e}c$ (for a recombination rate c per bp), to units of centimorgans per bp: $cM=50\ln\;\frac{1}{1\;-\;2c}$. As expected, recombination rate was negatively correlated with chromosome length (supplementary fig. S18, Supplementary Material online). Quadratic models fit to our data and those from Martin et al. calculated for *Heliconous* butterflies showed similar parameters, and the range of recombination rates overlapped with those measured in both *Heliconius* and *Drosophila* (Chan et al. 2012; Martin et al. 2019).

### Modelling Selection on *CYP333B3*

Taylor et al. (2021) genotyped *CYP333B3* from *H. zea* specimens sampled in the United States at in 1998, 2002, and 2017, reporting an increase in the proportion of individuals with a derived genotype over time. We were therefore interested to know whether our estimate of $\hat{s}\;$ could explain these independently estimated shifts in the derived allele frequency, *q*. Starting from an observed initial allele frequency of $q_{1998}=$ 0.1, we modeled the change in allele frequency in each subsequent generation as


$$\Delta p=\frac{\;pq\;.\;[\;p(w_{AA}-w_{Aa})\;+\;q(w_{Aa}-w_{aa})]}{\bar{w}}$$

Here the reference genotypic fitness is homozygous ancestral, i.e.


$$w_{AA}=1$$

$$w_{Aa}=1+h\hat{s}$$

$$w_{aa}=1+\hat{s}$$

So the mean fitness is


$$\bar{w}=p^{2}w_{AA}+2pqw_{Aa}+\;q^{2}w_{aa}$$

We predicted allele frequencies through time assuming a dominant-acting *CYP333B3* mutation (*h* = 1; Fig. 7b) and assuming codominance (*h* = 0.5; supplementary fig. S19, Supplementary Material online) for a range of generation times between 2 and 10 generations per year. This was repeated for both the lower and upper estimates of the selection coefficient, $\hat{s}$. The biologically realistic range of generation times in this case is unlikely to be below 5 generations/yr (Hardwick 1965; Parajulee et al. 2004; Morey 2012).

Additionally, we compared our mean estimate $\hat{s}=$ 0.049 to the selection coefficient that best explained the allele frequencies observed by Taylor et al*.,*  ${\hat{s}}_{\;fit}\;$. To estimate ${\hat{s}}_{\;fit}$, we ran 10^6^ iterations of the model with a random selection coefficient, retaining the coefficient that maximized the fit to the data. Fit was quantified as 1 minus the absolute difference between the observed and expected allele frequencies for years where allele frequencies were measured. This was done assuming complete dominance (*h* =1), assuming codominance (*h* =0.5), and with a random dominance coefficient to identify the combination of selection and dominance coefficients that maximized the fit to the data (supplementary table S7, Supplementary Material online; supplementary fig. S20, Supplementary Material online). The model and optimization algorithm are available at the Github repository listed under “Data Availability”.

We emphasize that our aim was to determine whether our estimate of the selection coefficient was biologically plausible, i.e. whether it was in the ballpark of values that could explain independently observed allele frequency estimates. This approach is not appropriate for determining a precise estimate of the selection coefficient; four important caveats should be considered. First, we estimate the selection coefficient over hundreds of generations. Realistically, the strength of selection imposed by pesticide exposure will vary substantially over space and time. Since the effect of selection on allele frequency depends not only on its strength but on the standing frequency, our estimate will not necessarily reflect the average strength of selection over time. Second, we compare our retrodiction with empirical data that is itself only a course estimate of the actual allele frequency through time; Taylor et al. sampled between 22 and 52 chromosomes, so sampling error substantially affects the observed allele frequency. Third, only three timepoints are used, and since we model Δp from $q_{1998}$ (to avoid any assumptions about when and how the allele first arose), we are only able to compare our estimates to two timepoints. The timepoints are, however, conveniently spaced for distinguishing between the distinct allele trajectories of dominant versus recessive mutations.

The fourth and most important caveat is that our comparison of $\hat{s}$ and ${\hat{s}}_{\;fit}\;$ is qualitative as substantially different methods are used for these estimates. Durrett and Schweinsberg’s (2004) approximation of α do not take dominance into consideration. Maynard Smith and Haigh (1974) show that dominance affects the extent to which genetic diversity is reduced at neighboring sites through hitch-hiking, though this effect is still highly localized. Therefore, the estimate of *s* based on SweepFinder's α may be an over-estimation if the advantageous derived *CYP333B3* allele is completely dominant, and is not directly comparable with estimates of *s* that assume the advantageous allele is dominant. Allele frequencies modeled in Fig. 7b are based on $\hat{s}$, yet assume complete dominance of the derived allele, so they should not strictly be interpreted as expected allele frequencies. Supplementary fig. S19, Supplementary Material online shows expected allele frequencies assuming co-dominance and produces qualitatively similar results. Although assuming co-dominance may allow for a more consistent comparison, this assumption may be biologically unrealistic: many cases of cytochrome P450-mediated pesticide resistance involve dominance coefficients >0.5, and we expect a gain-of function mutation allowing xenobiotic metabolism to act in a dominant manner (see Discussion; Cariño et al. 1994; Heckel et al. 1998; Sayyed et al. 2008; Achaleke and Brévault 2010; Han et al. 2014). Moreover, ${\hat{s}}_{\;fit}\;$ was a substantially worse fit to the empirical data when codominance was assumed—the observed rate of change early in time was far more consistent with selection on a dominant mutation (supplementary table S7, Supplementary Material online). In this study at least, the difficult decision between directly comparable estimates and biologically realistic assumptions is resolved by the fact that ${\hat{s}}_{\;fit}\;$ does not vary by more than ∼0.05 for dominance coefficients between 0.5 and 1 (see supplementary fig. S, Supplementary Material online); differences between *s* estimates based on historical allele frequencies and estimates of *s* based on sweep parameters are similar irrespective of whether we assume the mutation is codominant, dominant, or anywhere in between. Therefore, despite these caveats, our estimate $\hat{s}$ is entirely consistent with independently measured shifts in allele frequency over time.

### Estimating *CYP333B3* Allele Frequency in 2019

To estimate the empirical frequency of the *CYP333B3* allele in all 237 *H. zea* samples from 2019, we used as SNP within the gene region as a proxy for the allele frequency, reasoning that SNPs within the gene region would be completely linked, given the size of the gene region, the rate of LD decay, and the observed shifts in allele frequency over the recent past. To choose the most informative proxy SNP, we identified SNPs within the gene region with no more than 25% missing genotype information across all samples. All 12 such SNPs were close to fixation across samples (frequency >0.95), and were therefore supported by very few genotype calls. So, to minimise the possibility of genotyping errors influencing our estimate, we chose the SNP with the lowest major allele frequency: HaChr13:3519165, for which genotype calls could be made for ∼87% of samples. The allele frequency at this site was 0.956, and the allele was fixed among samples collected in North Carolina. The regional allele frequencies and genotype sample sizes are reported in supplementary table S6, Supplementary Material online.

### Testing for Cryptic Signatures of Adaptive Introgression at *CYP333B3*

We used three metrics to confirm that the sweep at *CYP333B3* was due to recent selection within *H. zea* and not the result of adaptive introgression from *H. armigera*. Adaptive introgression should result in decreased genetic differentiation from the donor species and an increased time to the most recent common ancestor (TMRCA) between homologous alleles at sites abutting the selected locus, and a decrease at the locus itself (Setter et al. 2020). By contrast, recent selection should result in an increase in genetic differentiation compared to samples collected earlier in time and a decreased TMRCA at the locus and linked sites. Therefore, we calculated $F_{ST}\;$ between *H.* zea samples from 2002 versus 2019, as well as $F_{ST}\;$ between *H. armigera* and *H. zea* sampled in 2019. This was done in both 20 kbp and 100 kbp windows. Next, we used Gamma-SMC (Schweiger and Durbin 2023) to estimate the TMRCA between homologous alleles at each polymorphic site on chromosome 13 within individuals sampled in 2019, using the average chromosome 13 scaled recombination rate estimate $\bar{\rho }=0.217$ as a prior. This was only done within individuals (as opposed to between alleles of different individuals) so that bias resulting from potential phasing errors could be ruled out. All such analyses were carried out using call set 1.

### Cross-Population Haplotype Homozygosity

We calculated the cross-population haplotype homozygosity (XP-EHH) (Sabeti et al. 2007) using the unphased implementation of *selscan2* to calculate the statistic using physical distance along chromosomes (as opposed to map distance) (Szpiech 2024). This was done using autosomal SNPs nonadmixed samples using call set 1, which were then imputed. The first test compared *H. zea* samples from 2002 collected by Taylor et al. (2021) to all 2019 *H. zea* samples (supplementary fig. S12 to S13, Supplementary Material online). The most significant peak was seen on chromosome 13 in a region overlapping with the *CYP333B3* locus (see Results and supplementary fig. S13, Supplementary Material online). Since the threshold of significance for the XP-EHH ratio is somewhat arbitrary, and given the other lines of evidence for a selective sweep at *CYP333B3*, we used this peak to define a threshold for subsequent tests (XP-EHH > 1.5 or <1.5). To test for regionally specific sweeps within the 2019 data, we grouped samples into four geographic regions: North Carolina, Texas, Missouri, and samples from either Arkansas or Louisiana. We calculated XP-EHH by comparing samples from each region to the remainder of 2019 samples (supplementary figs. S14 to S17, Supplementary Material online).

## Supplementary Material

msae129_Supplementary_Data

## Data Availability

All raw .fastq files generated here are available on the Short Read Archive: biosample accessions SAMN27502736-SAMN27502972, along with call sets as .vcf files. All custom scripts, processed data and scripts required to reproduce figures, and supplementary tables, are available at https://github.com/hlnorth/north_american_helicoverpa_zea.
