# Supplementary material for: Rapid Adaptation and Interspecific Introgression in the North American Crop Pest Helicoverpa zea
Source: Mol Biol Evol. 2024 Jun 28;41(7):msae129. doi: 10.1093/molbev/msae129 (PMC11259193; doi:10.1093/molbev/msae129)
Supplement: msae129_Supplementary_Data [file msae129_supplementary_data.zip › North_etal_supplementary_materialsMBE-23-0805.R1.pdf]

Supplementary Materials

Supplementary Figures

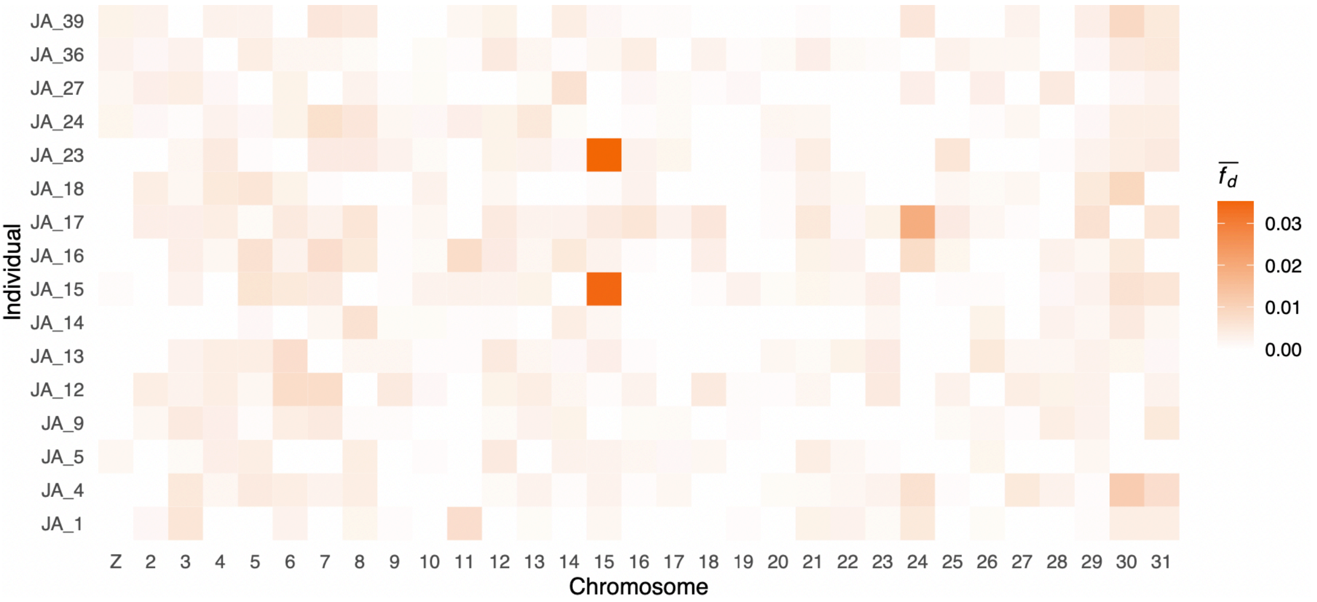

Supplementary Figure S1: Allele sharing on chromosome 15 in two individuals. Distribution of $\widehat{f_d}$ calculated in 20kbp windows where P1: *H. zea* sampled in 2002, P3: *H. armigera*, outgroup: *H. punctigera*. The statistic was calculated 16 times for each individual sampled in Jackson County, TX in 2019.

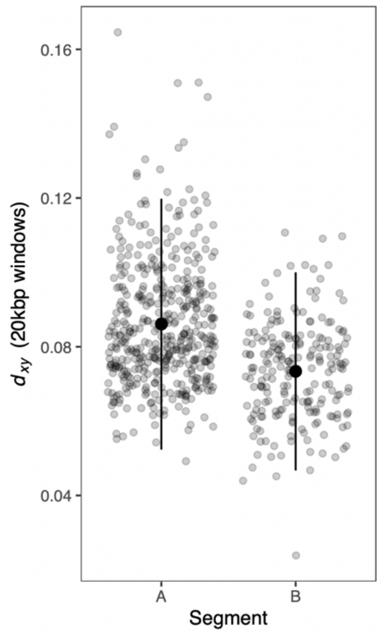

Supplementary Figure S2: Lower genetic divergence on segment B . Genetic divergence (d_{xy}), calculated in 20kbp windows, between the admixed individuals and *H. armigera* in the chromosomal segments labelled in Figure 3B.

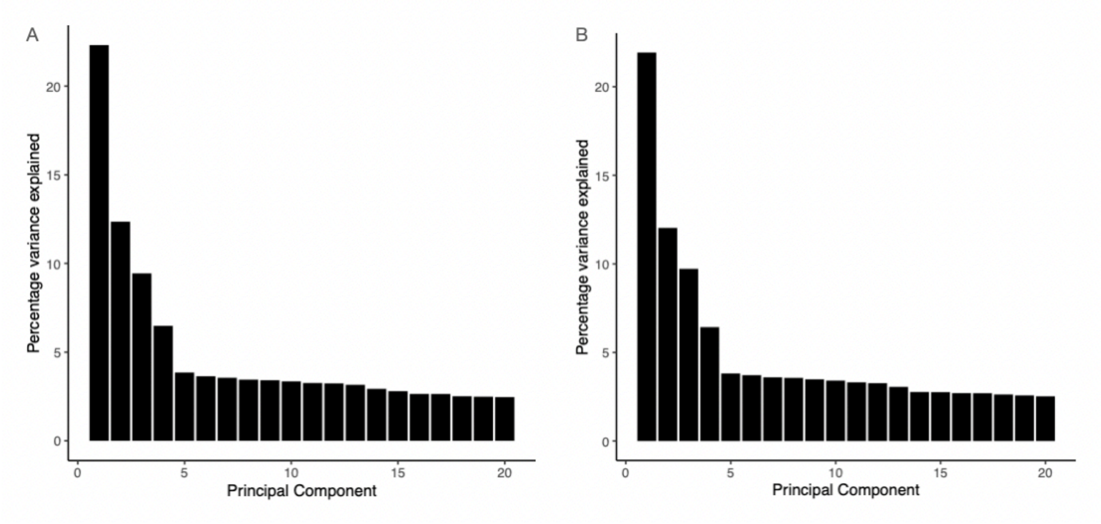

Supplementary Figure S3: Proportion of variance explained by each principal component. Data presented separately for chromosomal segments A and B, respectively, as show in Figure 4.

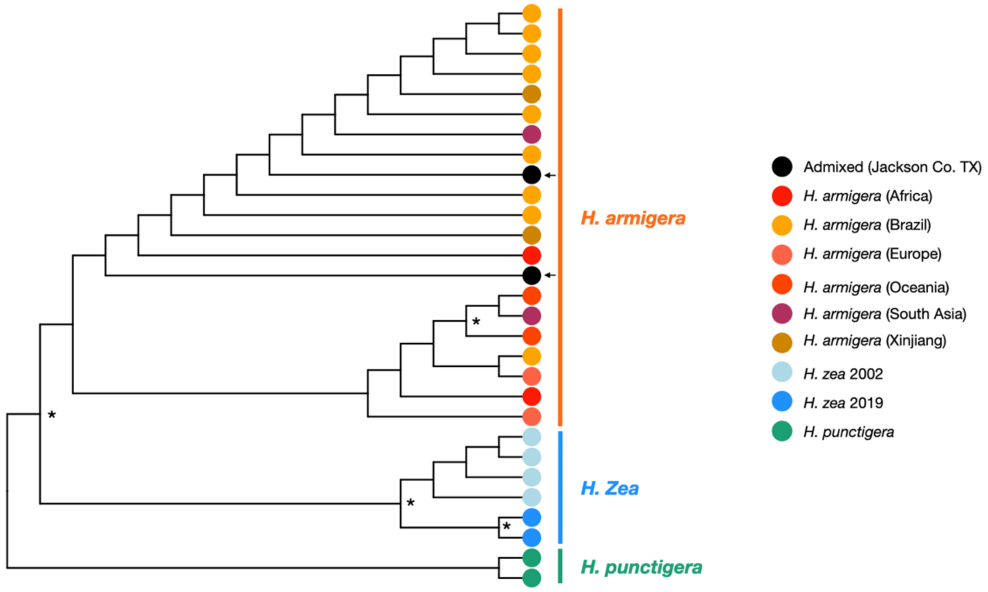

Supplementary Figure S4: At the *CYP337B3* locus, admixed samples form a clade with *H. armigera* samples. Maximum likelihood cladogram rooted with *H. punctigera* samples. Asterixis denote nodes with >95% bootstrap support. Arrows indicate admixed samples. Horizontal lines group species-level clade. There was consistent bootstrap support separating species-level clades and *H. zea* samples from 2002 versus 2019, but not within *H. armigera*. *H. zea* samples collected in 2002 are from Taylor *et al.* (2021). Samples from other *Helicoverpa* species are form Anderson *et al.* (2018) and Jin *et al.* (2023); see Supplementary Table S2.

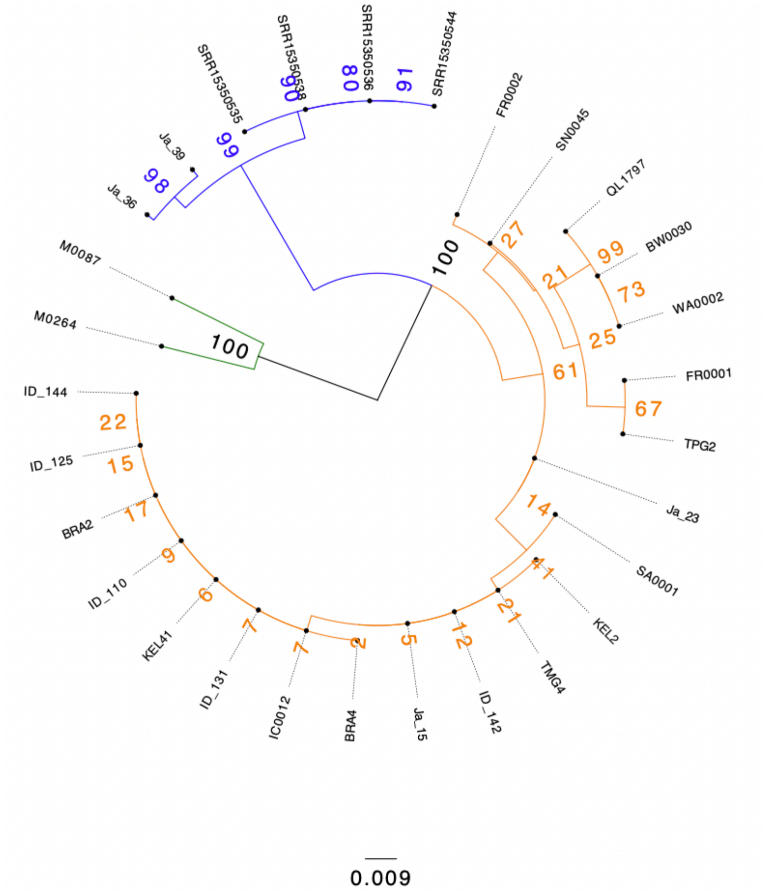

Supplementary Figure S5: Phylogram of *CYP337B7*. Tip labels indicate sample ID (described in Supplementary Table S2) and numbers indicate bootstrap values. This phylogram corresponds to cladogram shown in Supplementary Figure S4.

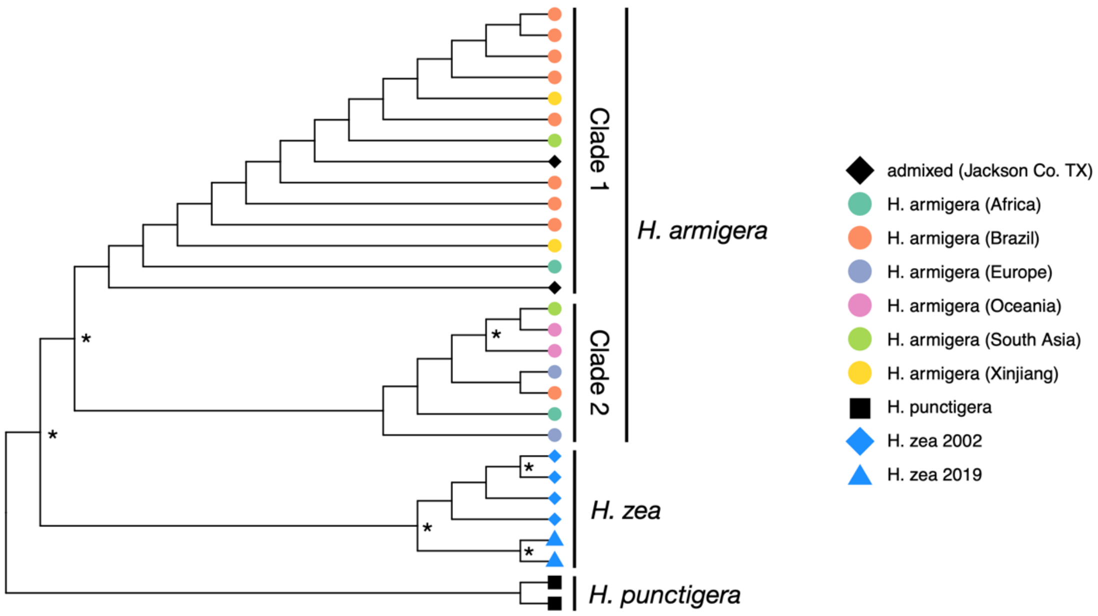

Supplementary Figure S6: Cladogram of *CYP337B7* with a constrained topology. Asterixis denote nodes with >95% bootstrap support. Arrows indicate admixed samples. Horizontal lines group species-level clade.The same tree as Figure S4, produced using a constrained topology requiring [(armigera_clade, zea_clade), punctigera_clade]. Clade 1 is now strongly supported and likely to be the *CYP337B3* allele under selection in Brazil.

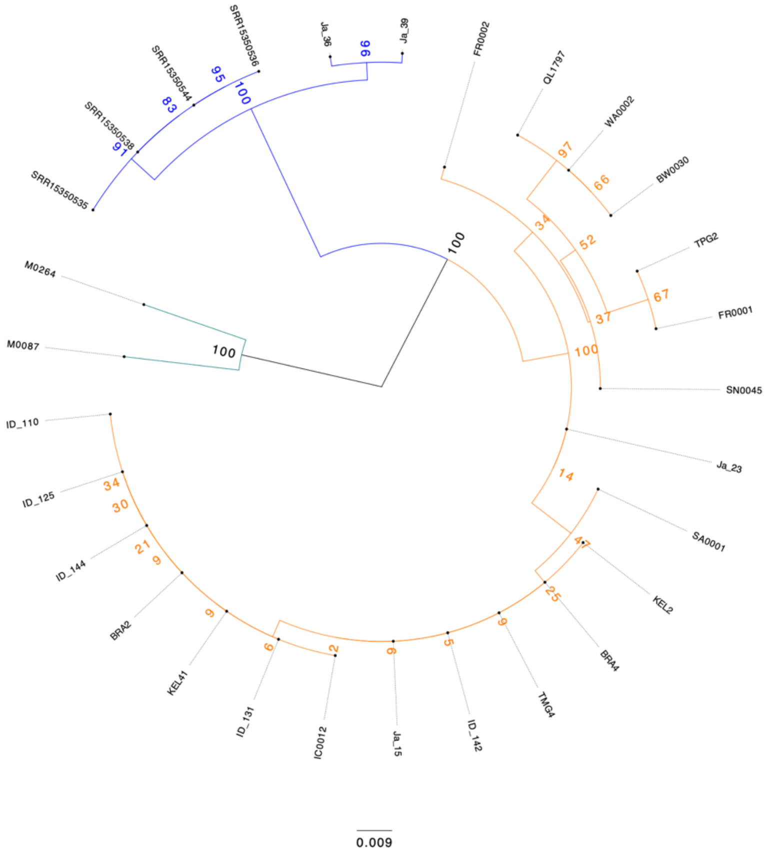

Supplementary Figure S7: Phylogram of *CYP337B7* with a constrained topology. Tip labels indicate sample ID (described in Supplementary Table S2) and numbers indicate bootstrap values. This phylogram corresponds to cladogram shown in Supplementary Figure S6.

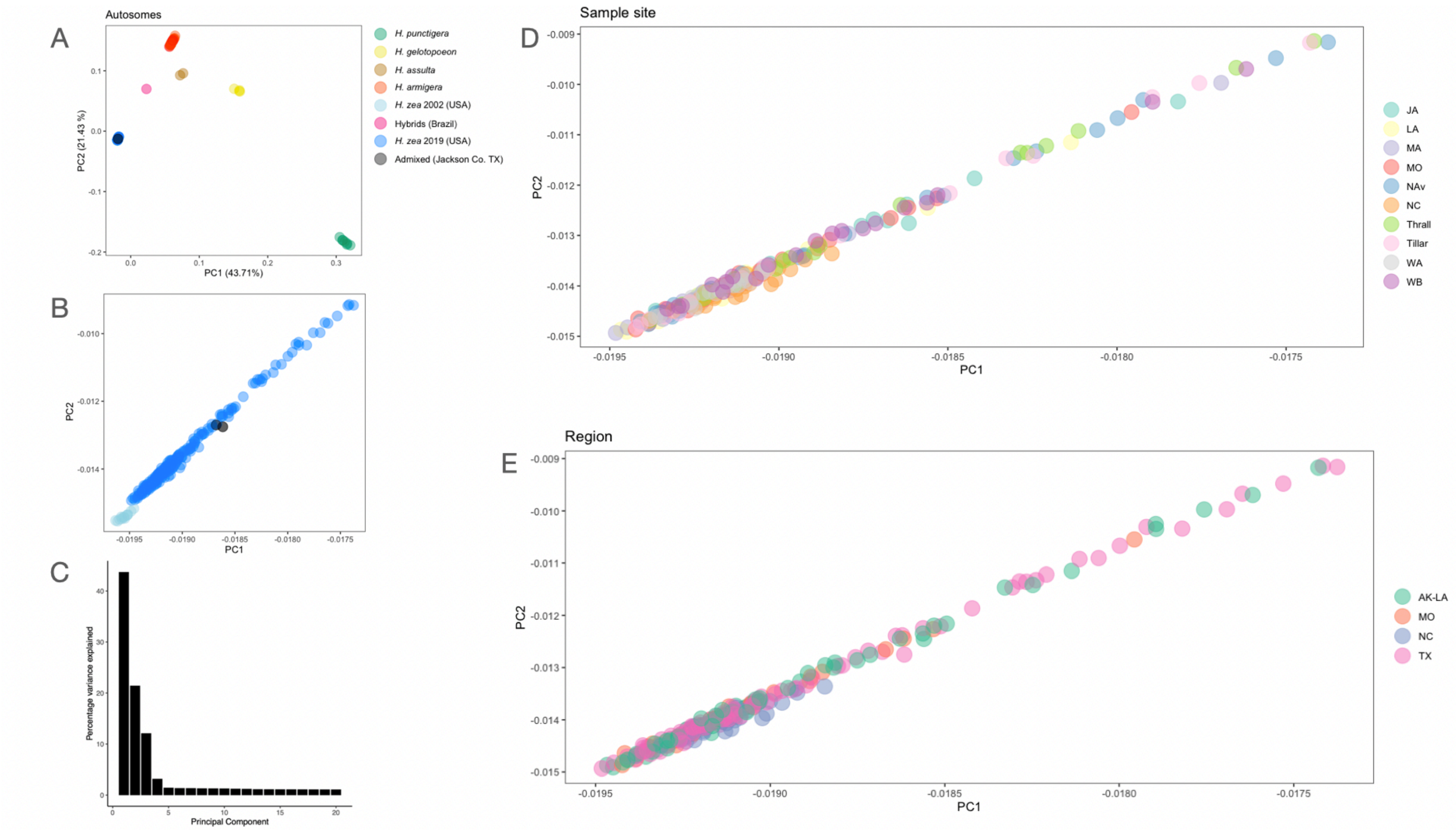

Supplementary Figure S8: Principal components analysis based on autosomal SNPs. A: All samples. **B**: *H. zea* samples only. **C**: Proportion of variance explained by each principal component. **D**: *H. zea* samples collected in 2019, coloured by sample site. **E**: *H. zea* samples collected in 2019, coloured by geographic region.

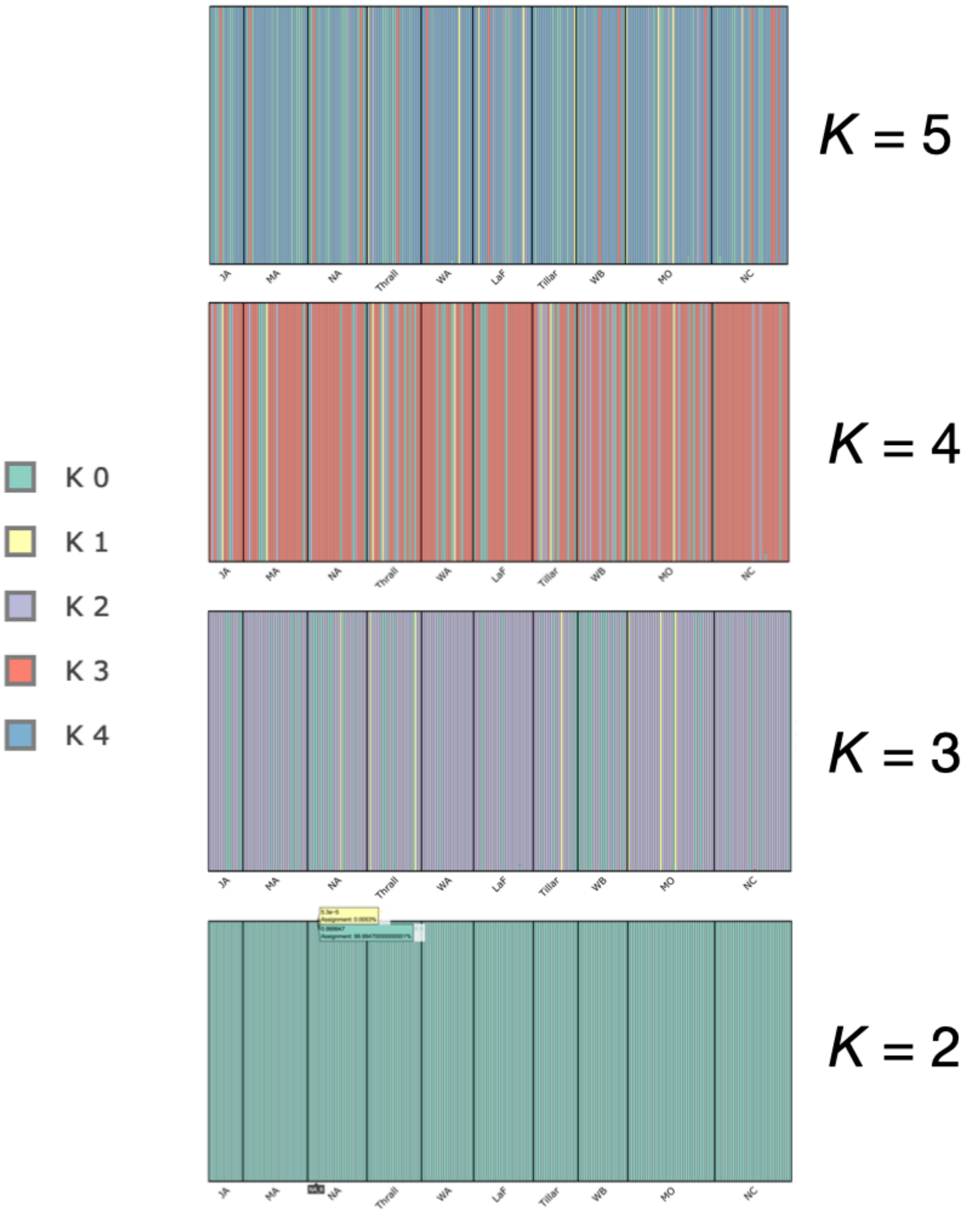

Supplementary Figure S9: ***fastStructure*** **plots for** ***K*****<5.** The value of K that maximised the marginal likelihood and best explained structure was K=1. For sub-optimal values of *K*>1, there is no correspondence between the sample site and the cluster assignment. Note that where *K*=2, individuals were assigned to K1 with proportions <0.01, which are too small to be shown in the figure. Proportions for sample NA_6 are highlighted as an example.

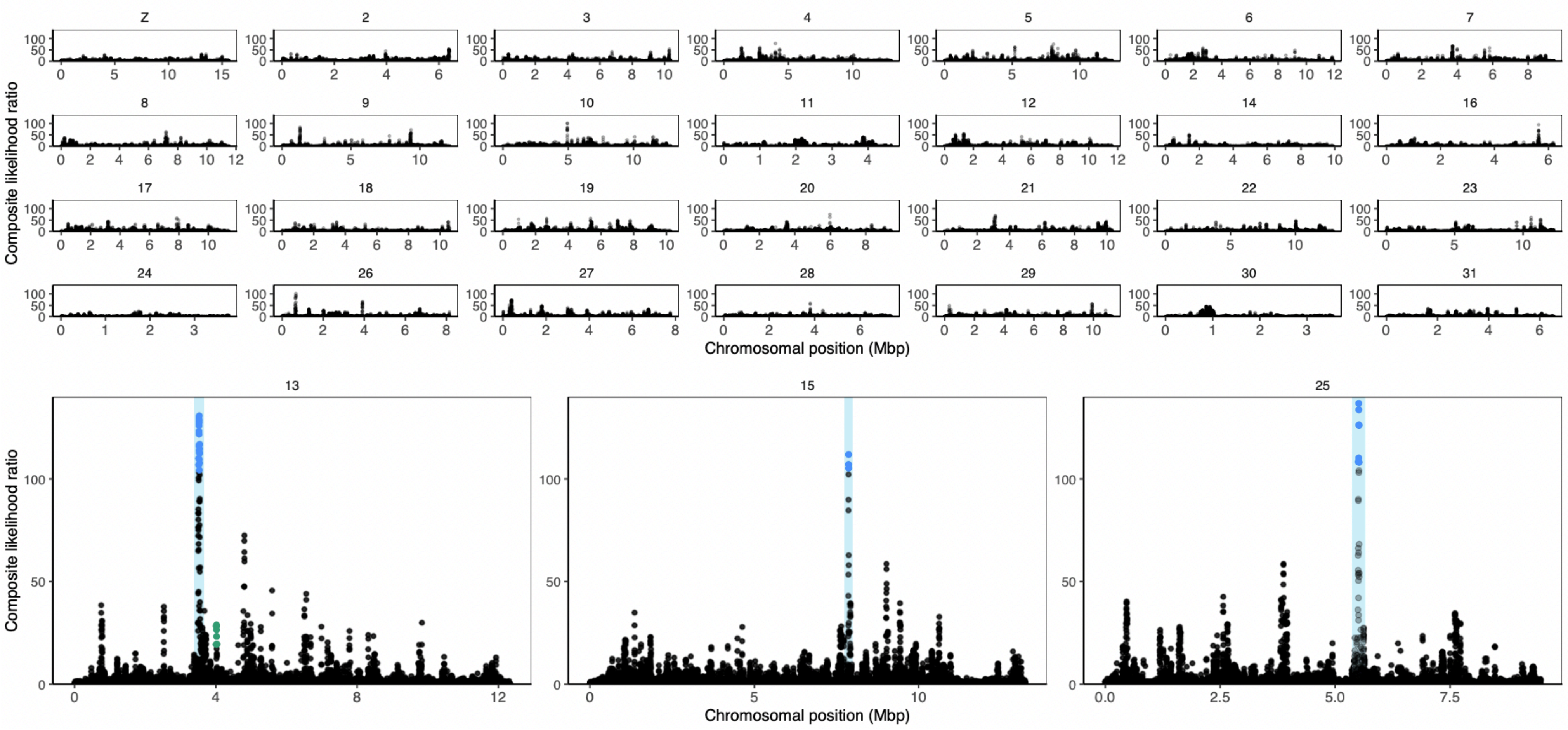

Supplementary Figure S10: Selective Sweep composite likelihood ratio (CLR) for each chromosome. Three chromosomes, on which selective sweeps were identified, are enlarged and highlighted. Sites in blue are in the upper 0.01st percentile of CLR values. Regions highlighted in blue shown in Figures 7 and 8. Points in green are in the upper 1st percentile and occur within the candidate Bt gene *PIK3C2A/kinesin-12*-like shown in Figure 9.

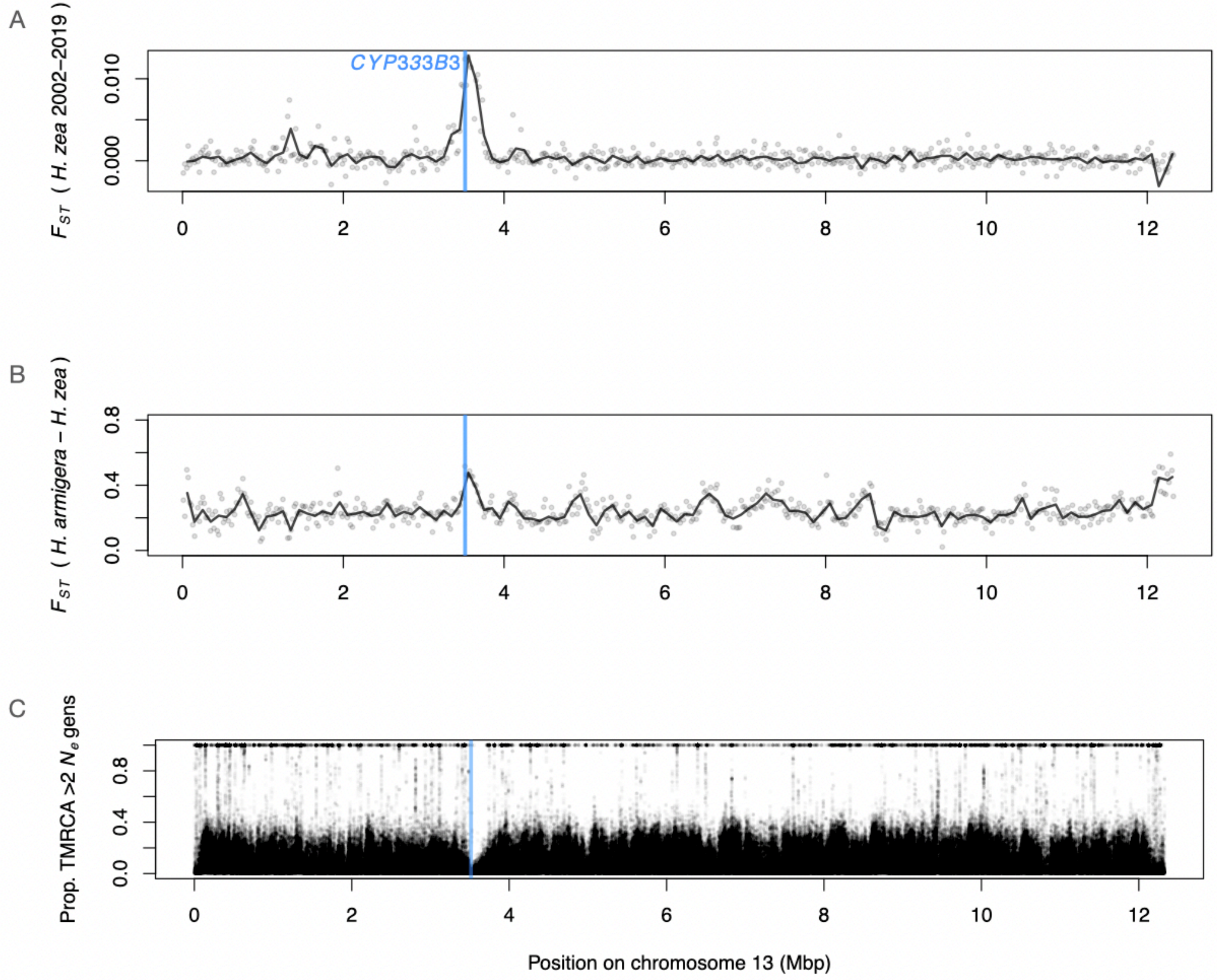

Supplementary Figure S11: Evidence for a recent selective sweep, and against introgression, on chromosome 13. A: Genetic differentiation (F_{ST}) between *H.* zea samples collected in 2002 vs. those collected in 2019, calculated in 20kbp windows (points) and 100kbp windows (lines) along chromosome 13. Blue line indicates the position of *CYP333B3*. **B:** F_{ST} between *H.* zea samples collected in 2019 vs. *H. armigera*. **C:** For each polymorphic site, the proportion of *H. zea* samples collected in 2019 with an estimated time to the most recent common ancestor (TMRCA) greater than $2N_e$generations in the past between homologous alleles. Samples from 2002 were collected by Taylor *et al.* (2021).

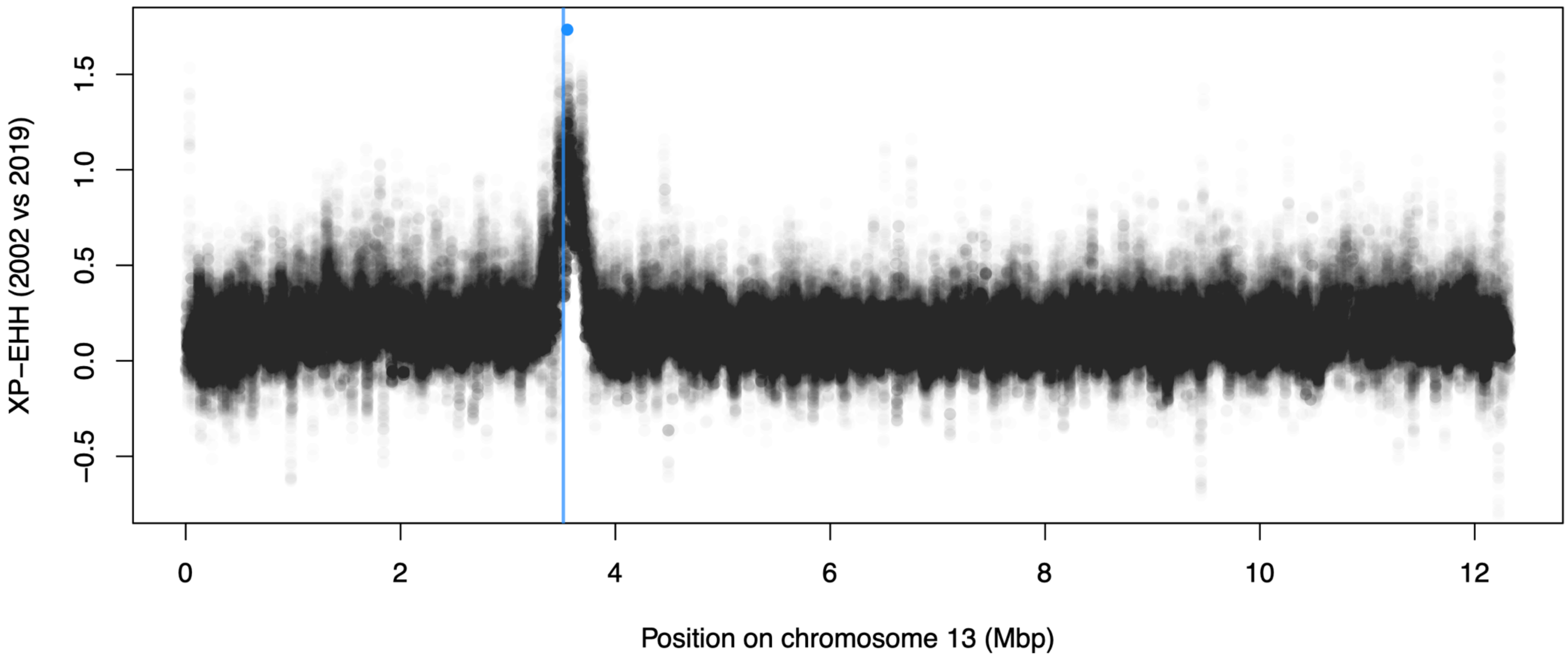

Supplementary Figure S12: Further evidence of a recent selective sweep at *CYP333B3*. Cross-population extended haplotype homozygosity (XP-EHH) comparing *H. zea* samples from 2002 and *H. zea* samples from 2019. Blue line indicates the centre of the *CYP333B3* locus. Blue point is the highest XP-EHH value. Positive values indicate a relative excess of long homozygous haplotypes in the 2019 samples compared to the 2002 samples.

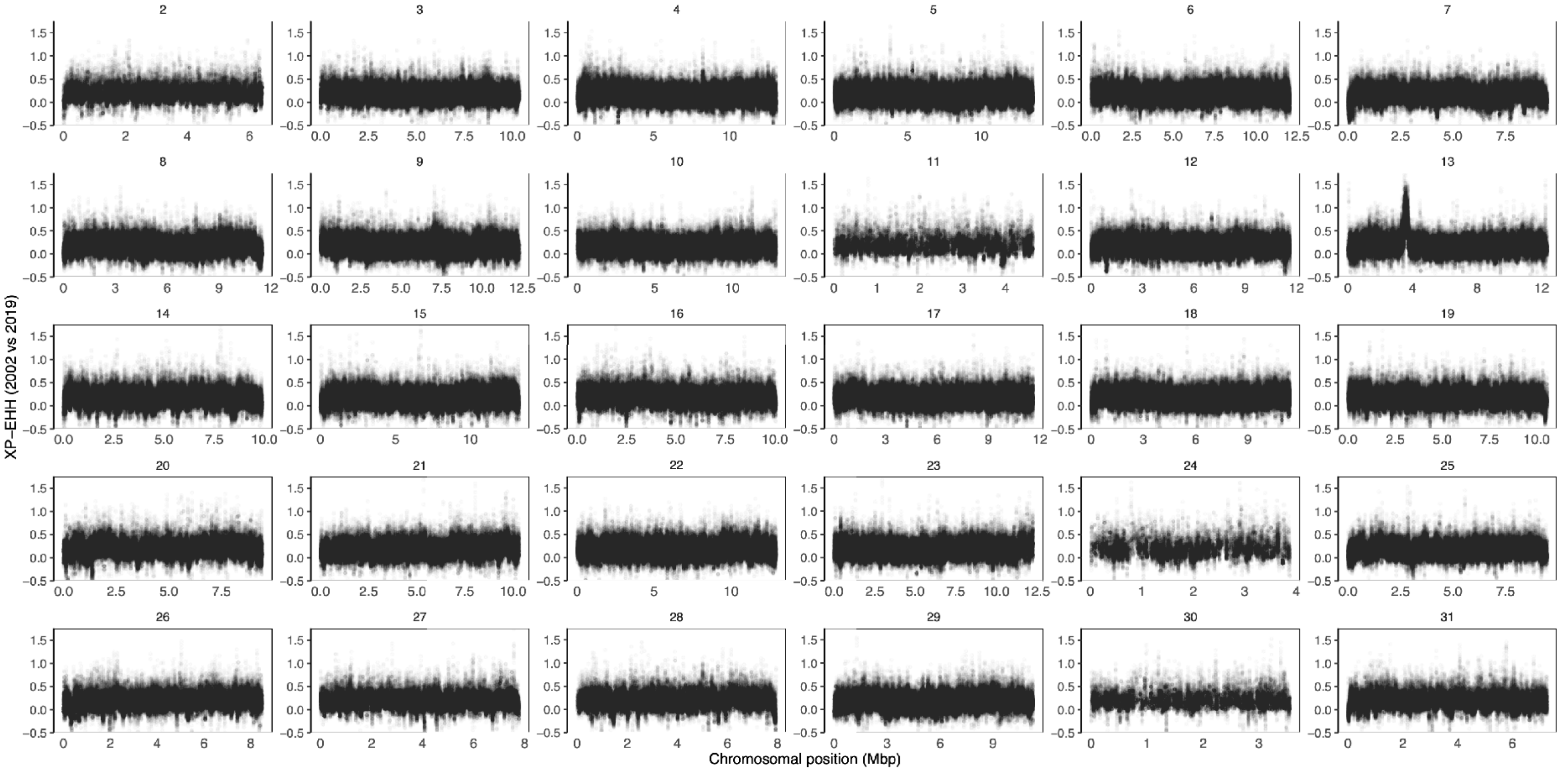

Supplementary Figure S13: XP-EHH Comparing 2002 and 2019 *H. zea* samples. Cross-population extended haplotype homozygosity (XP-EHH) comparing *H. zea* samples from 2002 and *H. zea* samples from 2019.

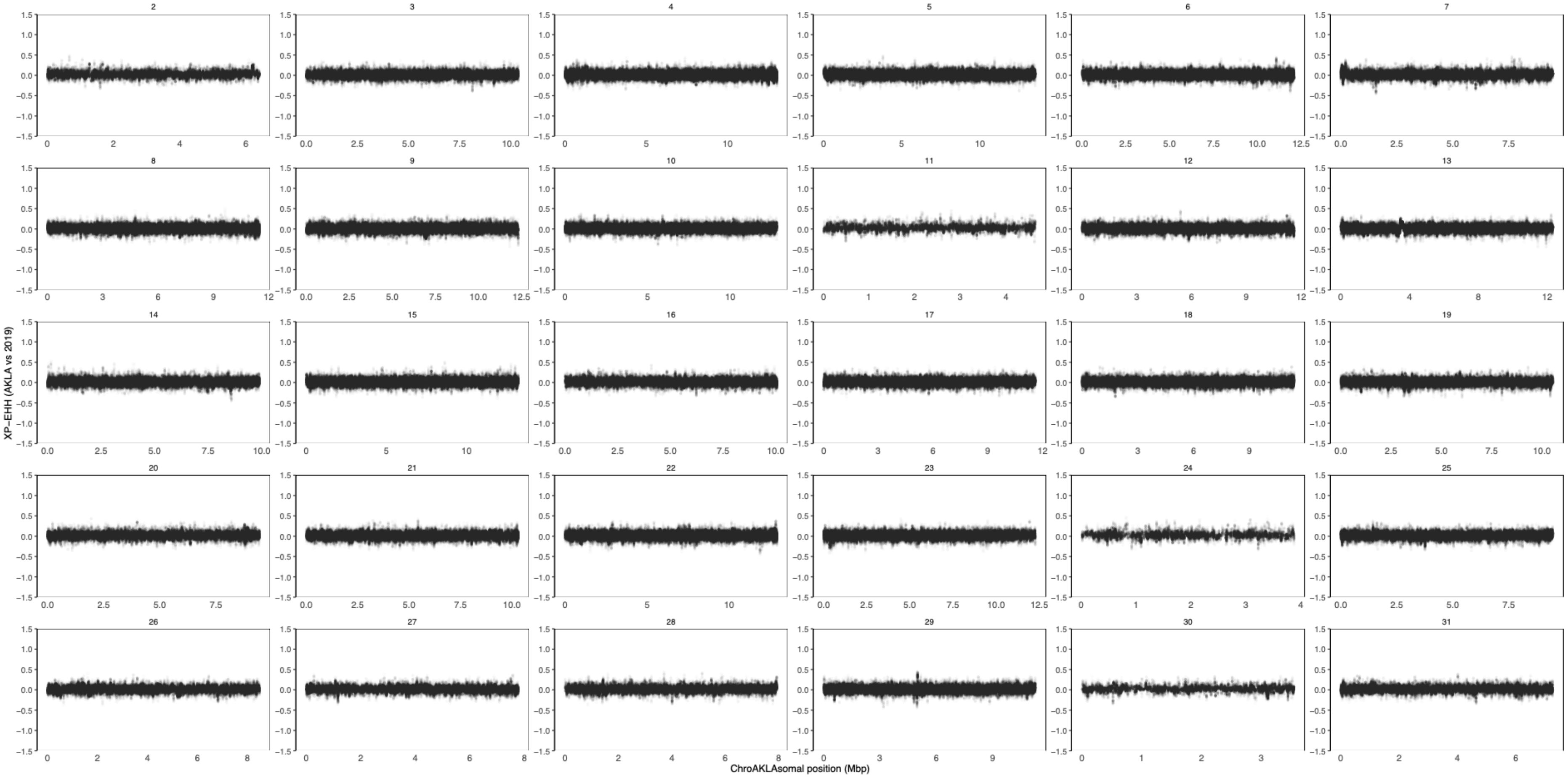

Supplementary Figure S14: XP-EHH Comparing samples from Arkansas and Illinois to all other 2019 *H. zea* samples. Results presented for each autosome.

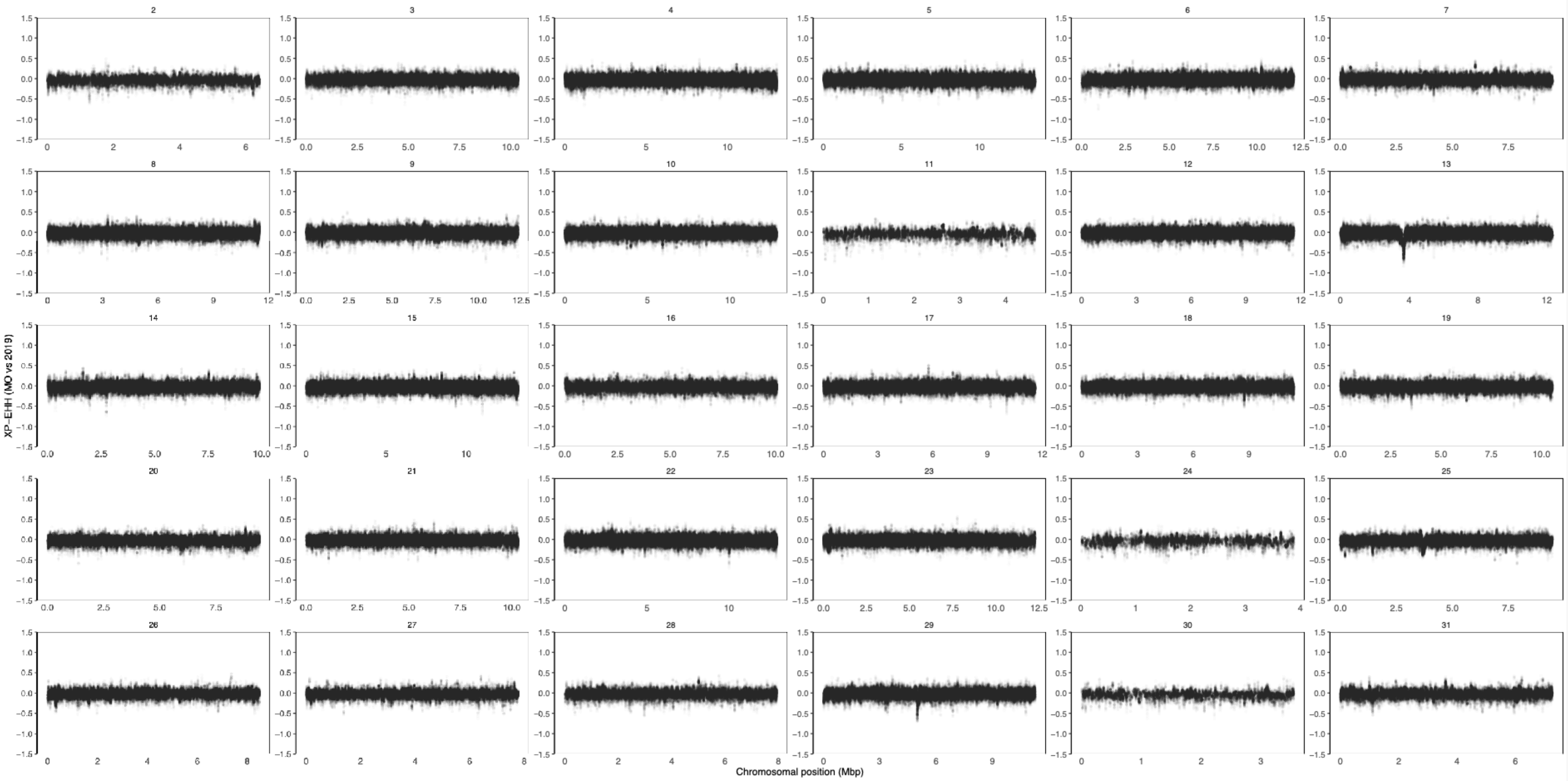

Supplementary Figure S15: XP-EHH Comparing Missouri samples to all other 2019 *H. zea* samples. Results presented for each autosome.

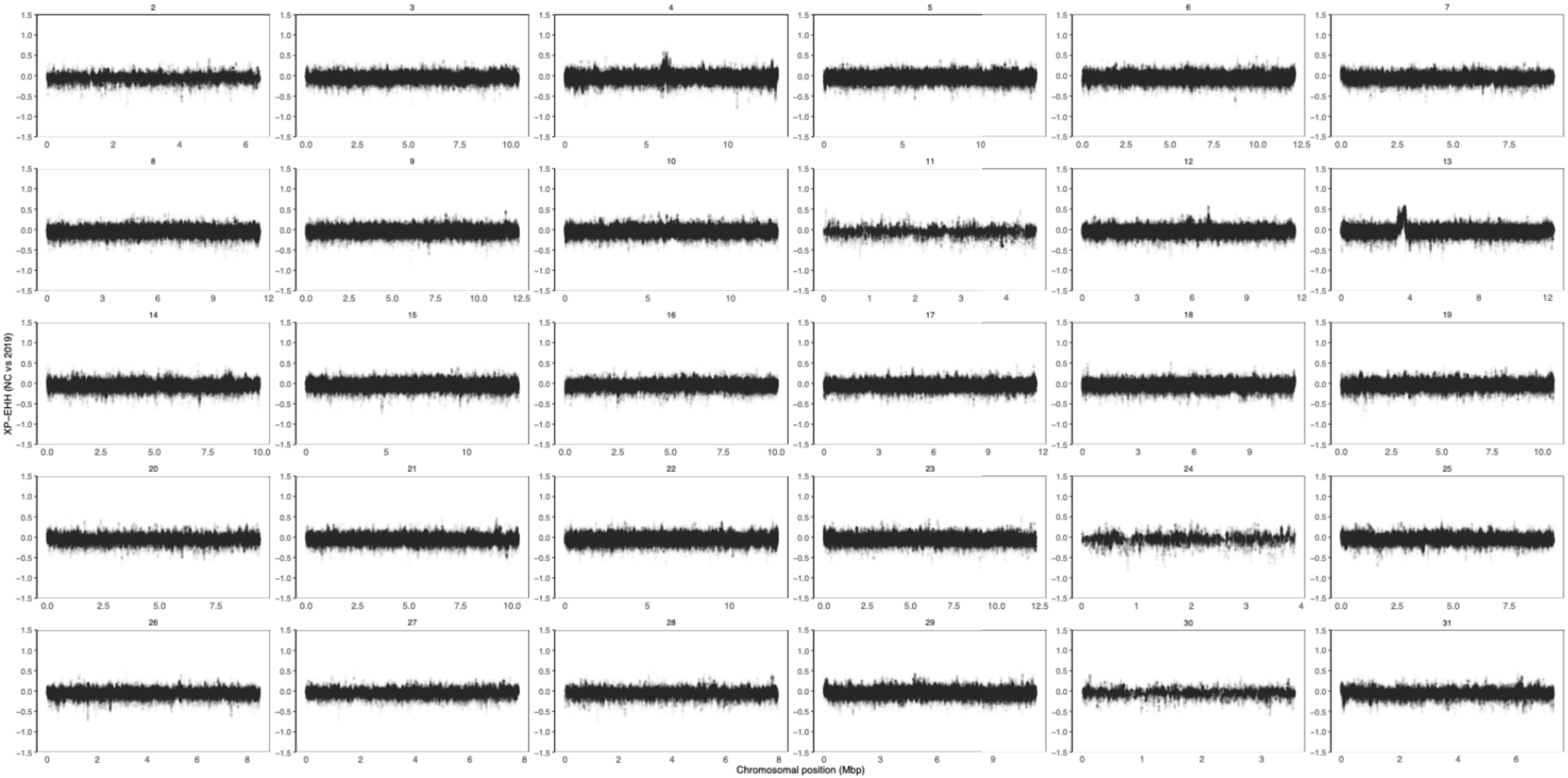

Supplementary Figure S16: XP-EHH Comparing North Carolina samples to all other 2019 *H. zea* samples. Results presented for each autosome.

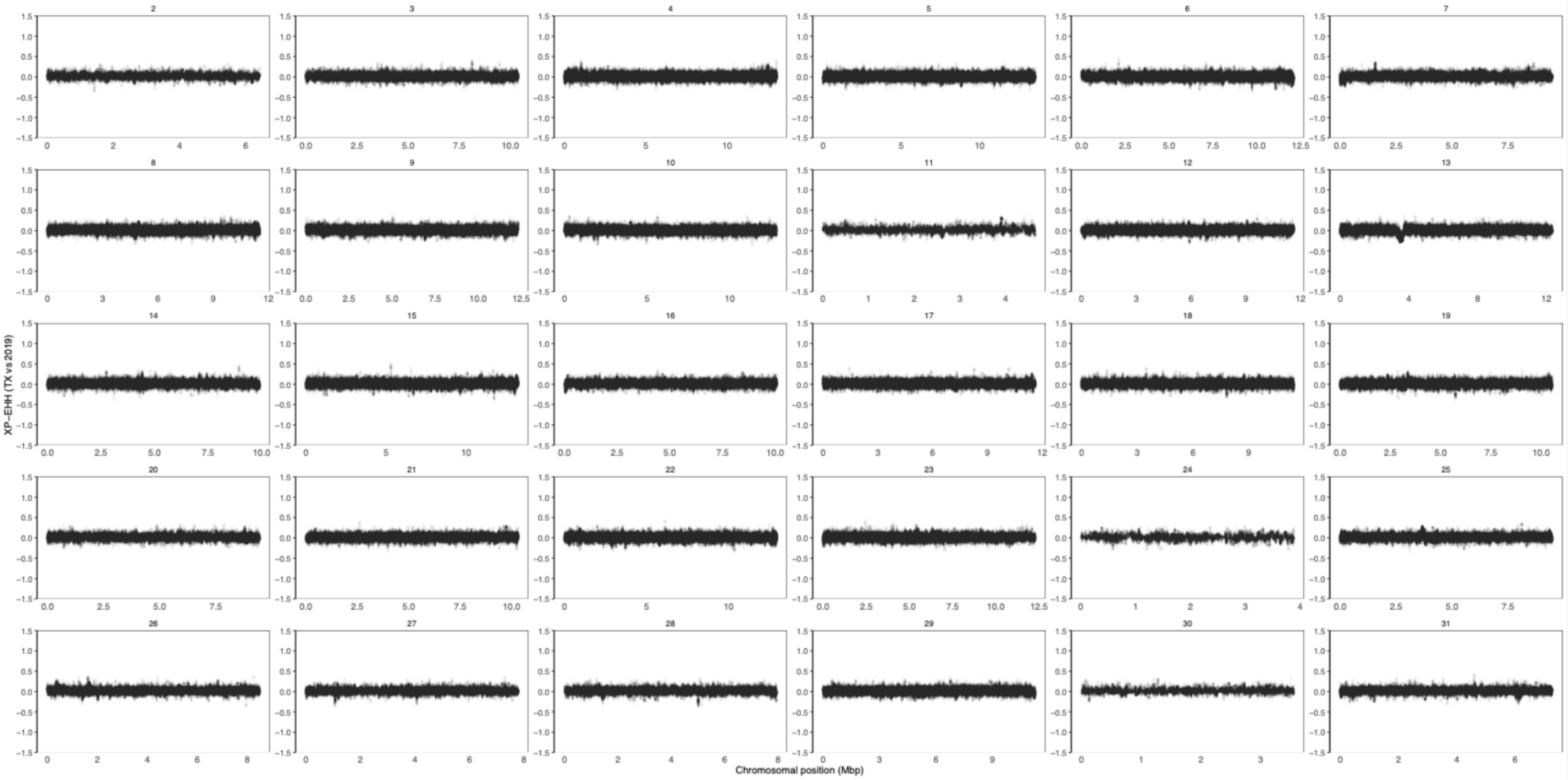

Supplementary Figure S17: XP-EHH Comparing Texas samples to all other 2019 *H. zea* samples. Results presented for each autosome.

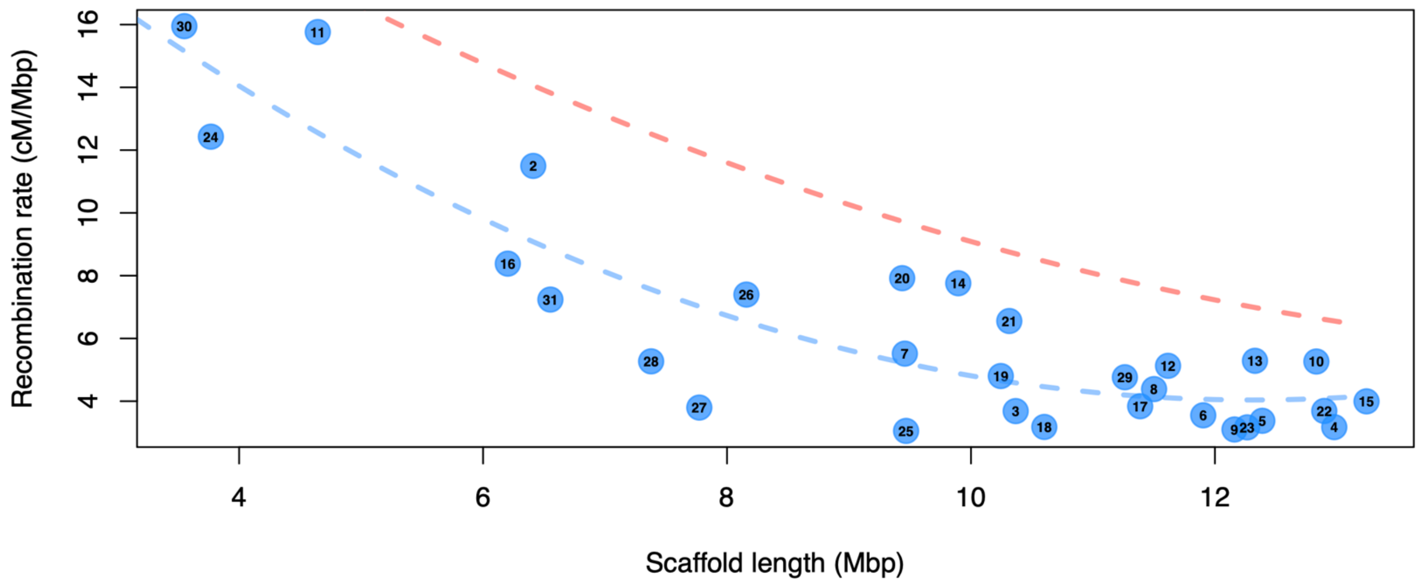

Supplementary Figure S18: Greater estimated mean recombination rate on shorter chromosomes. Per-chromosome recombination rate estimates against scaffold size. Blue dashed line shows quadratic model fit to data presented here. Red dashed line shows model fit to data from *Heliconius* butterflies generated by Martin *et al.* (2019).

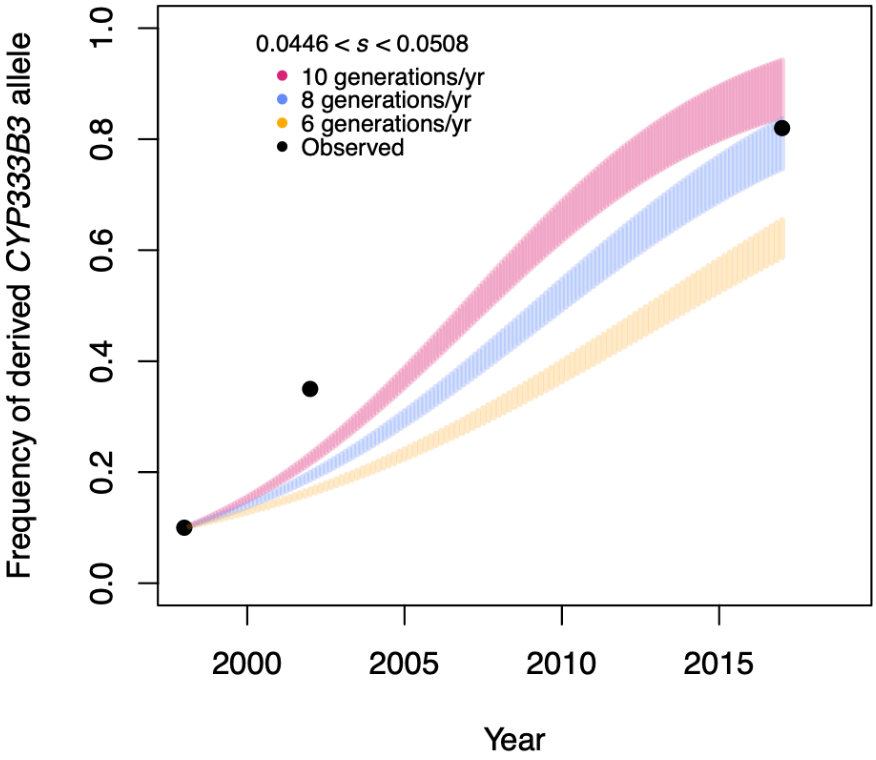

Supplementary Figure S19: Retrodicted allele frequencies assuming codominance. Predicted frequency of a codominant derived CYP333B3 allele for each generation given the estimated selection coefficient at that locus assuming 6, 8 and 10 generations per year. For each generation, a vertical line extends from the retrodicted allele frequency under the lower estimate of the selection coefficient ($\hat{s}$ =0.0446) to that estimated under the upper estimate ($\hat{s}$ = 0.0508). Line colours correspond to assumed generation time.

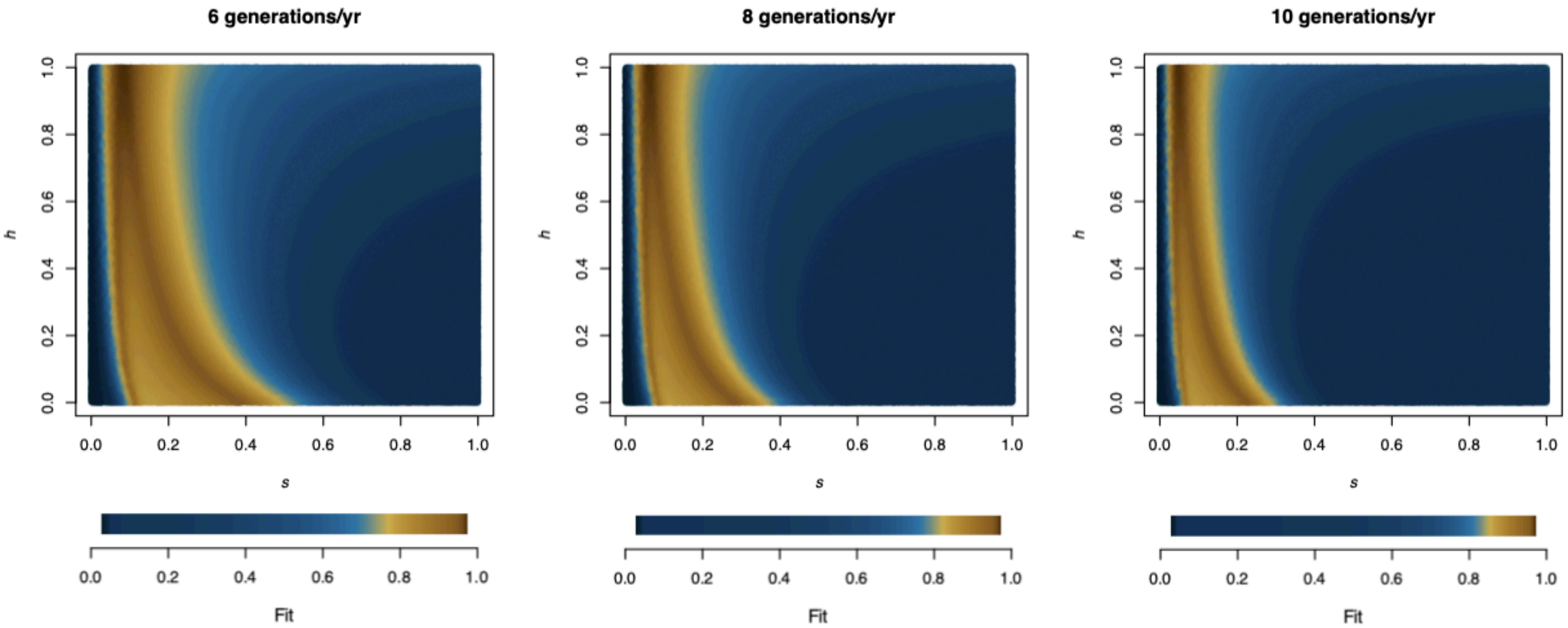

Supplementary Figure S20: Estimates of the selection coefficient, dominance coefficient and number of generations that best explain observed allele frequencies of *CYP333B3*. Our model of selection was run 10^6 iterations of the model with a random selection coefficient (s), dominance coefficient (h) and one of three possible generation times. For each, the model fit was calculated. Model fit was quantified as 1 minus the absolute difference between the observed and expected allele frequencies for years where allele frequencies were measured. The parameter values that maximised fit are reported in Supplementary Table S6.

Supplementary Tables

All supplementary tables are also available as text files, and at https://github.com/hlnorth/north_american_helicoverpa_zea

Supplementary Table S1: Sample sites

ID	location_raw	lat	lon	sample_count	source	year
TX-Ja	Jackson, Co., Texas (2019)	28.94	-96.58	16	K. Crumley	2019
TX-Ma	Matagorda Co., Texas (2019)	28.78	-96	26	K. Crumley	2019
TX-Na	Navasota, Texas (2019)	30.383333	-96.083333	24	D. Kerns	2019
TX-Th	Thrall, Texas (2019)	30.588611	-97.298611	22	D. Kerns	2019
TX-Wh	Wharton, Texas (2019)	29.311667	-96.102778	21	K. Crumley	2019
AR-La	Lafayette Co., Arkansas (2019)	33.264167	-93.592778	24	G. Lorenz	2019
AR-Ti	Tillar, Arkansas (2019)	33.711389	-91.453056	18	G. Lorenz	2019
LA-Wb	Winnsboro, Louisiana (2019)	32.163333	-91.723333	20	S. Brown	2019
MO-Mi	Mississippi Co., Missouri (2019)	36.83	-89.29	35	X. Shirley	2019
NC-LM	Lees Mill Township, NC (2019)	35.8205262	-76.633798	31	D. Reisig	2019

Supplementary Table S2: Sample metadata

See text file and https://github.com/hlnorth/north_american_helicoverpa_zea

Supplementary Table S3: Gene annotations in sweep regions

gene_name	chromosome	start	end	strand	annotation_source	annotation_ID	annotation_name
CYP333B3	13	3515066	3521020	-	Scipio	HaOG200024	CYP333B3_Ha
uncharacterised protein	13	3526715	3531067	-	EVM	HaOG204154	LOC101736753BMORI:uncharacterized protein LOC101736753
Cpq-like	13	3550470	3552580	-	EVM	HaOG204153	BMORI:carboxypeptidase Q-like
TARDB43-like	15	7845568	7847853	+	EVM	HaOG201405	BMORI:TAR DNA-binding protein 43-like
RNF186-like	15	7854240	7857883	-	EVM	HaOG201406	BMORI:E3 ubiquitin-protein ligase RNF168-like
Hyd-like	15	7858581	7878070	+	EVM	HaOG201407	BMORI:E3 ubiquitin-protein ligase hyd-like
PAQR3-like	15	7879195	7881223	-	EVM	HaOG201408	BMORI:progestin and adipoQ receptor family member 3-like
toX2-like	15	7882653	7884625	+	EVM	HaOG201409	BMORI:protein takeout-like isoform X2
clock-like	15	7885820	7887805	-	EVM	HaOG201410	BMORI:circadian clock-controlled protein-like
NAA40	25	5481491	5482145	-	EVM	HaOG206021	BMORI:N-alpha-acetyltransferase 40-like
AR-like	25	5482716	5485238	+	EVM	HaOG206020	BMORI:aldose reductase-like
Mcm5-like	25	5485331	5490182	-	EVM	HaOG206019	BMORI:DNA replication licensing factor Mcm5-like
dynein-like_1	25	5490930	5513636	+	EVM	HaOG206018	BMORI:dynein beta chain, ciliary-like
dynein-like_2	25	5513930	5522191	+	EVM	HaOG206017	BMORI:dynein beta chain, ciliary-like
inversin-A-like	25	5522550	5530858	-	EVM	HaOG206016	BMORI:LOW QUALITY PROTEIN: inversin-A-like
UCP	25	5538018	5540668	-	EVM	HaOG206015	BMORI:uncharacterized protein LOC101745936

Supplementary Table S4: Candidate Bt gene coordinates and permutation test statistics

name	chr	chr_start	chr_end	mean_CLR	mean_CLR_percentile	max_CLR	pval_meanLR	pval_maxLR	permutations
HGSNAT	13	3972151	3990195	1.09265783	0.76662745	4.09238	0.268	0.294	1000
liph-b	13	4007535	4012428	2.175093	0.86089749	6.267544	0.156	0.098	1000
PIK3C2A	13	4012595	4041462	10.4048056	0.9744208	28.879544	0.024	0.017	1000
kinesin-12	13	4017085	4018787	15.896281	0.9865347	19.368941	0.006	0.002	1000
hz_G0000111	13	4042941	4043929	4.198217	0.92454249	4.198217	NA	NA	NA
hz_G0000112	13	4046460	4054376	1.88253588	0.84322895	5.255188	0.181	0.145	1000
UBE3A	13	4055437	4064654	3.7718144	0.91605822	7.390298	0.086	0.109	1000
PDE6D	13	4065371	4066829	5.169933	0.93927816	5.657602	0.066	0.058	1000
apn1	9	905656	913026	0.2207265	0.5277424	0.875827	0.683	0.643	1000
apn4	9	916818	923372	0.0675095	0.38013618	0.261402	0.899	0.87	1000
white	10	10530120	10553467	2.50175829	0.8768614	6.243568	0.135	0.213	1000
tspan1	10	10636587	10646992	1.8538855	0.84132987	7.403905	0.179	0.119	1000
cad_86C	12	3443579	3504322	0.69541972	0.69338661	9.173318	0.479	0.265	1000
map4K4	15	6352455	6365131	0.55843623	0.66039583	2.14861	0.46	0.486	1000
abcC2	15	6465002	6476633	4.18051636	0.92422165	13.601682	0.075	0.048	1000
Cry1Ab_KZ118765	9	6911296	6919881	0.08620925	0.39921444	0.238912	0.889	0.913	1000
Cry1A105_Cry2Ab2_KZ118015	9	10037688	10052829	0.28438907	0.56254152	1.111046	0.679	0.749	1000
Cry1Ab_NW_018395566	9	6711005	6755334	1.83118051	0.83967709	8.825868	0.181	0.207	1000
Cry1A105_Cry2Ab2_NW_018395399	9	8191879	8338153	0.5715646	0.66394444	10.793561	0.656	0.363	1000

Supplementary Table S5: Nonsynonymous genotypes identified in *Kinesin*-12. Position refers to nucleotide in the full gene sequence including untranslated regions and noncoding regions.

pos	WT_allele	alternate_allele	WT_AA	alternate_AA	samples_hom	samples_het	samples_alt_hom	samples_uncallable	samples_called	obs_et	exp_het	obs_hom_derived	exp_hom_derived	alt_allele_frew	allele_freq
1130	C	A	Q	K	99	1	0	137	100	0.01	0.00995	0	0.000025	0.005	0.995
1374	C	A	T	K	75	20	1	138	96	0.20833333	0.20290799	0.01041667	0.01312934	0.11458333	0.88541667
1239	C	T	S	L	91	10	0	136	101	0.0990099	0.09410842	0	0.00245074	0.04950495	0.95049505
900	A	G	Q	R	92	5	0	140	97	0.05154639	0.05021788	0	0.00066426	0.0257732	0.9742268
1314	A	C	D	A	98	4	0	135	102	0.03921569	0.03844675	0	0.00038447	0.01960784	0.98039216
911	A	T	S	C	94	2	1	140	97	0.02061856	0.04038686	0.01030928	0.00042513	0.02061856	0.97938144
1186	T	A	N	K	95	3	0	139	98	0.03061225	0.03014369	0	0.00023428	0.01530612	0.98469388
926	G	A	D	N	90	2	0	145	92	0.02173913	0.02150284	0	0.00011815	0.01086957	0.98913044
881	G	A	D	N	90	2	0	145	92	0.02173913	0.02150284	0	0.00011815	0.01086957	0.98913044

Supplementary Table S6: Allele frequency at the *CYP333B3* locus among 235 *H. zea* samples collected in 2019.
Allele frequencies are reported for the SNP HaChr13:3519165

Sample grouping	All 2019 samples	Texas	Missouri	North Carolina	Arkansas and Louisiana
**Individual sample size**	237	109	35	31	62
**Individuals genotyped (%)**	86.49789	82.56881	94.28571	87.09677	88.70968
**Allele frequency**	0.9560976	0.9277778	0.969697	1	0.9727273

Supplementary Table S7: Estimates of the selection coefficient from observed data match estimates inferred from the sweep assuming biologically realistic generation times. $\hat{s}_{fit}$ was estimated (1) when it was allowed to co-vary with the dominance coefficient, (2) assuming complete dominance, and (3) assuming co-dominance. This was repeated for different generation times (2-10 generations per year). Each estimate is based on 10^6 iterations of the model. The mean estimate of the selection coefficient from the selective sweep was 0.04894071. Biologically realistic generation times are 8-10 generations/yr. The error score is the absolute difference between the observed and expected allele frequencies for years where allele frequencies were measured. The 'fit' value in Supplementary Figure S20 is one minus the error score.

Generations per year	s_fit_variable_h	h_fit	error_score_variable_h	s_fit_complete_dominance	error_score_complete_dominance	s_fit_codominance	error_score_codominance
2	0.2857278	0.9999344	0.02323998	0.2870514	0.02273232	0.2146638	0.151069
3	0.182458	0.99954	0.02581397	0.1827668	0.02543528	0.1386256	0.1522696
4	0.1337418	0.9999447	0.02701145	0.1340026	0.026774	0.1023504	0.1528644
5	0.1058976	0.9971798	0.02866044	0.105763	0.02757429	0.08112028	0.1532273
6	0.08732526	0.998097	0.0289676	0.08735047	0.02810524	0.06718035	0.1534518
7	0.07375967	0.9993274	0.02972536	0.0743956	0.02848393	0.05732962	0.1536213
8	0.06446656	0.9981957	0.03010817	0.06478627	0.02876881	0.04999753	0.1537447
9	0.0572161	0.9992821	0.02961346	0.05737369	0.02898859	0.0443284	0.1538422
10	0.05143565	0.9982418	0.03004393	0.05148375	0.02916437	0.0398144	0.1539259

Citations (supplementary materials)

Anderson CJ et al. 2018. Hybridization and gene flow in the mega-pest lineage of moth, Helicoverpa. Proceedings of the National Academy of Sciences. 201718831.

Jin M et al. 2023. Adaptive evolution to the natural and anthropogenic environment in a global invasive crop pest, the cotton bollworm. The Innovation. 4:100454.

Martin SH, Davey JW, Salazar C, Jiggins CD. 2019. Recombination rate variation shapes barriers to introgression across butterfly genomes. PLoS Biol. 17:e2006288.

Taylor KL, Hamby KA, DeYonke AM, Gould F, Fritz ML. 2021. Genome evolution in an agricultural pest following adoption of transgenic crops. Proc Natl Acad Sci USA. 118:e2020853118.
